# Cancer EV stimulate endothelial glycolysis to fuel protein synthesis via mTOR and AMPKα activation

**DOI:** 10.1002/jev2.12449

**Published:** 2024-07-13

**Authors:** Joël E. J. Beaumont, Lydie M. O. Barbeau, Jinzhe Ju, Kim G. Savelkouls, Freek G. Bouwman, Marijke I. Zonneveld, Annelies Bronckaers, Kim R. Kampen, Tom G. H. Keulers, Kasper M. A. Rouschop

**Affiliations:** ^1^ Department of Radiotherapy, GROW‐School for Oncology and Reproduction Maastricht University Medical Centre+ Maastricht The Netherlands; ^2^ Department of Human Biology, NUTRIM School of Nutrition and Translational Research in Metabolism Maastricht University Medical Centre+ Maastricht the Netherlands; ^3^ Department of Cardio & Organ Systems (COS), Biomedical Research Institute Hasselt University Diepenbeek Belgium; ^4^ Laboratory for Disease Mechanisms in Cancer Department of Oncology, KU Leuven Leuven Belgium; ^5^ Leuven Cancer Institute (LKI) Leuven Belgium

**Keywords:** angiogenesis, extracellular vesicles, hypoxia, metabolism

## Abstract

Hypoxia is a common feature of solid tumours and activates adaptation mechanisms in cancer cells that induce therapy resistance and has profound effects on cellular metabolism. As such, hypoxia is an important contributor to cancer progression and is associated with a poor prognosis. Metabolic alterations in cells within the tumour microenvironment support tumour growth via, amongst others, the suppression of immune reactions and the induction of angiogenesis. Recently, extracellular vesicles (EV) have emerged as important mediators of intercellular communication in support of cancer progression. Previously, we demonstrated the pro‐angiogenic properties of hypoxic cancer cell derived EV. In this study, we investigate how (hypoxic) cancer cell derived EV mediate their effects. We demonstrate that cancer derived EV regulate cellular metabolism and protein synthesis in acceptor cells through increased activation of mTOR and AMPKα. Using metabolic tracer experiments, we demonstrate that EV stimulate glucose uptake in endothelial cells to fuel amino acid synthesis and stimulate amino acid uptake to increase protein synthesis. Despite alterations in cargo, we show that the effect of cancer derived EV on recipient cells is primarily determined by the EV producing cancer cell type rather than its oxygenation status.

## 1 INTRODUCTION

Reprogramming of energy metabolism is one of the hallmarks of cancer (Hanahan & Weinberg, 2011). In contrast to most non‐malignant cells, which prefer oxidative phosphorylation, a large subset of cancer cells rely on aerobic glycolysis for the production of ATP, despite its lower efficiency (two vs. 36 mole of ATP per mole of glucose) (Hanahan & Weinberg, 2011). The glycolytic pathway provides cancer cells with the carbon molecules required for the synthesis of amino acids, lipids, and nucleotides to sustain their high proliferative phenotype (Vander Heiden et al., 2009). In addition, high glycolytic flux leads to excessive lactate secretion and extracellular acidification which supports cancer progression via the induction of angiogenesis and inhibition of anti‐tumour immune reactions (Erra Díaz et al., 2018; Végran et al., 2011). Metabolic changes in non‐cancer cells present within the tumour microenvironment (TME) also contribute to tumour progression. For example, increased glycolytic flux in endothelial cells, triggered by angiogenic signals, modulates tip cell differentiation and is essential in the formation of new blood vessels (De Bock et al., 2013; Parra‐Bonilla et al., 2010; Yetkin‐Arik et al., 2019). In monocytes, glycolysis is crucial for their differentiation into macrophages and their subsequent polarisation into either the tumour‐suppressive M1 or tumour‐supportive M2 phenotypes (Hou et al., 2020; Palsson‐McDermott et al., 2015). Moreover, their glycolytic activity enables tumour‐associated macrophages to stimulate metastasis development (Penny et al., 2016). Cancer associated fibroblasts (CAFs) support tumour progression via the induction of angiogenesis, stimulation of invasion and metastasis, modulation of immune reactions and promoting therapy resistance (Joshi et al., 2021). Increased glycolytic activity in CAFs provides metabolites that fuel the pentose phosphate pathway and nucleotide metabolism of cancer cells (Becker et al., 2020). Hence, inducing glycolysis in cancer and tumour‐resident non‐malignant cells contributes to cancer progression. Cellular glucose metabolism and alterations herein are tightly controlled by AMP‐activated protein kinase α (AMPKα) and mammalian target of rapamycin (mTOR) mediated signalling (Battaglioni et al., 2022; Herzig & Shaw, 2018; Park et al., 2017; Torrence et al., 2021).

AMP‐activated protein kinase α (AMPKα) plays a crucial role in maintaining cellular energy homeostasis via stimulation of glucose catabolism (Herzig & Shaw, 2018). Upon its activation through phosphorylation of Thr17, AMPKα stimulates glucose uptake and glycolytic flux through activation of glucose transporters and glycolytic enzymes (Herzig & Shaw, 2018). The mammalian target of rapamycin (mTOR) is a serine/threonine protein kinase which forms two distinct protein complexes, mTOR complex 1 (mTORC1) and mTOR complex 2 (mTORC2) (Battaglioni et al., 2022). These complexes phosphorylate a plethora of proteins involved in transcription, translation and metabolism (Battaglioni et al., 2022). Eukaryotic translation initiation factor 4E‐binding protein 1 (4E‐BP1) binds and inhibits eIF4E, thereby inhibiting the initiation of protein translation (Battaglioni et al., 2022). Phosphorylation of 4E‐BP1 by mTORC1 at Thr37, Thr46, Ser65 and Thr70 reduces its affinity for eIF4E and allows the initiation of protein translation (Battaglioni et al., 2022). Moreover, mTORC1 phosphorylates ribosomal protein S6 kinase (RPS6K) at Thr389, which in turn phosphorylates various substrates that drive protein production, including eukaryotic translation initiation factor 4B and eukaryotic elongation factor 2 kinase (Battaglioni et al., 2022; Magnuson et al., 2012).

Extracellular vesicles (EV) are nano‐sized membrane vesicles containing a variety of proteins, nucleic acids and lipids which are secreted by most cell types (Raposo & Stoorvogel, 2013). In recent years, EV have gained great interest from the scientific community because of their pathophysiological role in intercellular communication in several diseases, including neurodegenerative‐ and autoimmune diseases and cancer (Meehan & Vella, 2016; Vanherle et al., 2020; Wen et al., 2016; Xavier et al., 2020; Yáñez‐Mó et al., 2015). Cancer cells secrete EV to manipulate surrounding cells to create a growth‐promoting environment. EV include exosomes and microvesicles, which are generated through distinct biogenesis pathways (Raposo & Stoorvogel, 2013). Exosomes are formed through inward budding of the endosomal membrane and are subsequently released via fusion of the multivesicular endosome with the plasma membrane, whereas microvesicles bud directly from the plasma membrane (Raposo & Stoorvogel, 2013). Although the biogenesis of both types occurs at different subcellular locations, they share various intracellular mechanisms and sorting machineries (van Niel et al., 2018). The overlap in size and cellular machineries complicates the distinction between EV subtypes after secretion and specific markers for each subtype are still lacking (van Niel et al., 2018; Welsh et al., 2024). Exposure of EV producing cells to environmental factors such as hypoxia alters the molecular composition, content and downstream effects of EV (Beaumont et al., 2022; Keulers et al., 2021; Walbrecq et al., 2020; Walbrecq et al., 2020; Zonneveld et al., 2019).

Tumour hypoxia, caused by the continuous proliferation of cancer cells and defective vasculature, is a common feature of solid tumours and is associated with poor survival independent of treatment modality (Brown & Wilson, 2004; Harris, 2002). Hypoxia activates cellular survival mechanisms including autophagy and the unfolded protein response, resulting in therapy resistance and a more aggressive phenotype (Brown & Wilson, 2004; Harris, 2002; Rouschop et al., 2010). Moreover, hypoxia triggers profound alterations in lipid‐, amino acid‐ and glucose metabolism (Infantino et al., 2021). It increases the expression of glycolytic enzymes and reduces delivery of NADH and FADH_2_ to the electron transport chain, thereby shifting cellular metabolism towards glycolysis (Infantino et al., 2021). During hypoxia exposure, cancer cells secrete specific subpopulations of EV with a distinct molecular cargo (Keulers et al., 2021; Kucharzewska et al., 2013; Walbrecq et al., 2020a; Walbrecq et al., 2020b). These hypoxic cancer cell‐derived EV have been linked to the transfer of hypoxia tolerance to other cells within the TME and suppression of antitumor immune reactions (Beaumont et al., 2022; Zonneveld et al., 2019). Previously we demonstrated that hypoxic cancer cells stimulate angiogenesis via the secretion of EV (Keulers et al., 2021). In this study, we investigate the role of (hypoxic) cancer cell‐derived EV in the metabolic reprogramming of recipient cells. Our data indicate that cancer EV are important mediators of metabolic changes in recipient cells and induce a shift towards increased glycolysis. Although enhanced glycolysis is independent of the recipient cells type, in endothelial cells, the induced glucose consumption supports amino acid and nascent protein synthesis via mTOR and AMPKα activation. Interestingly, the enhanced glucose consumption is restricted to cancer cell derived EV only and is absent after exposure to normal cell derived EV. The observed effects are dependent on both the EV producing cancer cell type and their oxygenation status, suggesting that tumour cells secrete EV to manipulate their environment depending on their specific requirements. Our data indicate that EV‐mediated metabolic reprograming and induction of angiogenesis functions in parallel to the classical angiogenic pathways (e.g., soluble VEGF) and should be taken into consideration when developing novel anti‐angiogenic strategies.

## 2 MATERIAL AND METHODS

### 2.1 Cell culture

HT29 (colorectal adenocarcinoma), U87 (glioblastoma), MDA‐MB‐231 (breast adenocarcinoma), EC‐RF‐24 (immortalized human umbilical cord vascular endothelial cells (HUVECs)), HMEC‐1 (human microvascular endothelial cells), MRC5 (lung fibroblasts), U937 (monocytes) and BJ (fibroblasts) cells were cultured in DMEM (HT29, U87, MDA‐MB‐231, BJ—Sigma–Aldrich, D6429), RPMI (EC‐RF‐24, U937 ‐ Sigma–Aldrich, R8758), MCDB 131 (HMEC‐1, Gibco, 10372019) or EMEM (MRC5, Corning, 100‐009CV) supplemented with 10% foetal bovine serum (FBS) (Serana, S‐FBS‐SA‐015), 1 μg/mL hydrocortisone (HMEC‐1, Sigma–Aldrich, H0888), 1.25 μg/mL endothelial cell growth supplement (HMEC‐1, Sigma–Aldrich, E2759) and 2 mM glutamax (U937, HMEC‐1, Gibco, 35050–061) at 37°C in a humidified atmosphere containing 5% CO_2_. For endothelial cells, culture plates were coated with 0,2% gelatin (Sigma–Aldrich, G1393) in PBS for 10 min. Cells were routinely checked, and found negative, for mycoplasm contamination. Depletion of serum derived EV was performed by ultracentrifugation of 30% FBS in culture medium at 100,000 g for 16 h (Optima XPN‐90 Ultracentrifuge, Type 45 Ti rotor, Beckman Coulter) and filtration using a 0,22 μm polyethersulfone membrane filter (Corning, 431097). Before EV isolation, cells were seeded at a density of 22,500 cells/cm^2^ (HT29, U87, MDA‐MB‐231), 10,600 cells/cm^2^ (BJ), 50,000 cells/cm^2^ (MRC5), in standard culturing medium and allowed to attach for 24 h, after which they were washed with PBS and medium was changed for culturing media supplemented with 5% EV‐depleted FBS. U937 cells were seeded at a density of 800,000 cells/mL in EV depleted media. Cells were exposed to normoxia (21% O_2_), mild hypoxia (0,2% O_2_ – Whitley H85 hypoxystation—Don Whitley Scientific) or severe hypoxia (O_2 _< 0,02% ‐Whitley A35 anaerobic workstation—Don Whitley Scientific) for 24 h. To label EV delivered proteins, EV‐producing cells were cultured in methionine free DMEM (Gibco, 21013–024) supplemented with 5% EV depleted FBS, 2 mM GlutaMAX (Gibco, 35050–061), 1 mM sodium pyruvate (Gibco, 11360‐070), 0.0626 g/L L‐cysteine (Sigma–Aldrich, C1276) and 50 μM L‐Azidohomoalanine (L‐AHA, Invitrogen, C10102) during 24 h of EV collection. To evaluate the contribution of EV to the overall effect of the cancer cell secretome on recipient endothelial cell metabolism, HT29 cells were cultured in serum free medium and exposed to severe hypoxia. Subsequently, conditioned medium was EV depleted by ultracentrifugation at 100,000 g for 1 h.

### 2.2 EV isolation

Conditioned medium (CM) was collected after 24 h. Debris and contaminants were removed by differential centrifugation steps at 4°C (10 min 300 g, 20 min 2,000 g, 30 min 16,000 g—adapted from (Théry et al., 2006)). CM was then concentrated using Amicon Ultra‐15 Centrifugal Filter Units (Millipore, UFC910096) at 4000 g, 4°C to a final volume of 1 mL. EV were purified by size exclusion chromatography (SEC). In short, concentrated CM was loaded onto a 16 mL Sepharose CL‐2B column (Cytiva, GE17‐0140‐01) and eluted with PBS. Fractions of 1 mL were collected and the EV‐containing fractions 7 and 8 were pooled, aliquoted and stored at −80°C.

### 2.3 Protein quantification

Cells were lysed in RIPA buffer (50 mM Tris‐HCl, 150 mM NaCl, 0,1% Triton X100, 0,5% Sodium deoxycholate, 0,1% SDS, 1 mM sodium orthovanadate, 1 mM NaF, protease inhibitors (Roche Diagnostics, 11873580001), pH 8.0) for 30 min on ice. EV were lysed in RIPA buffer for 2 h on ice with regular vortexing. Subsequently, protein concentrations were determined using the Micro BCA Protein Assay Kit (for EV lysates, ThermoFisher scientific, 23235) or the Pierce BCA Protein Assay Kit (for cell lysates, ThermoFisher scientific, 23225) according to the manufacturer's instructions.

### 2.4 (Immunogold) cryo‐electron microscopy

HT29 derived vesicles were labelled with anti‐CD9 (BD Pharmingen, 555370), anti‐CD63 (BD Pharmingen, 556019) or anti‐CD81 (BD Pharmingen, 555675) antibodies for 30 min at room temperature (10 μg/mL, diluted in blocking buffer (10% bovine serum albumin in PBS)). Next, EV were pelleted by ultracentrifugation at 100,000 g for 60 min at 4°C (Optima XPN‐90 Ultracentrifuge, SW 41Ti rotor, Beckman Coulter), resuspended in blocking buffer and incubated with gold labelled anti‐mouse IgG (Aurion, 806.022) for 30 min at room temperature. Then, a thin aqueous film of the (un)labelled sample was formed on a Lacey Carbon Film (Electron Microscopy Sciences) by applying 2.5 μL of sample and blotting away excess liquid. The grid was held by tweezers in the environmental chamber (20°C and more than 95% relative humidity) of the Vitrobot mark IV (Iancu et al., 2006). After blotting, the grid with the thin aqueous film was rapidly vitrified by plunging into ethane cooled to its melting point (−180°C) by liquid nitrogen. The vitrified specimen was transferred to a cryo‐transmission electron microscope (Arctica 200KV) and pictures were taken with a Falcon III camera (adapted from (Keulers et al., 2021)).

### 2.5 Nanoparticle tracking analysis

Nanoparticle tracking analysis (NTA) was performed to evaluate particle size distribution and concentration of the EV isolates using the ZetaView Nanoparticle Tracking Analyzer (Particle Metrix GmbH—version 8.05.11 SP4). Before each use, the ZetaView was calibrated using 100 nm polystyrene beads (Particle Metrix GmbH, 110‐0020) according to the manufacturer's protocol. EV were diluted in PBS and measured in 11 different focus positions. Settings were kept constant for each acquisition and analysis: sensitivity 70, shutter 100, frame rate 30, minimal brightness 20, minimal area 5, maximal area 1000 and tracelength 30.

### 2.6 EV uptake

Endothelial cells were seeded on glass coverslips for confocal microscopy analysis or in a 24‐well culture plate for flow cytometric analysis and allowed to attach overnight. EV (and control PBS) were labelled using 5 μM carboxyfluoresceïne succinimidyl ester (CFSE) dye (Biolegend, 423801) for 20 min at 37°C. Next, cells were incubated with 1 μg/mL labelled EV (or equivalent volume of control PBS) for 6 h. For confocal imaging, cells were fixed using 4% paraformaldehyde for 20 min and nuclei were stained with Hoechst. Images were taken using a Leica TCS SP8 sted confocal microscope. Punctae per cell and the amount of CFSE‐positive cells were analyzed using ImageJ software. For flow cytometry, cells were trypsinized, washed with PBS containing 1% BSA and fluorescence intensity was measured using the BD FACS CANTO II. Data were analysed using FlowJo software (v10.8.1 FlowJo, Ashland, OR, USA).

### 2.7 Glucose uptake/lactate secretion

Endothelial cells (28,000 cells/cm^2^), cancer cells (31,000 cells/cm^2^) and fibroblasts (18,750 cells/cm^2^) were seeded and allowed to attach overnight. Adhered cells or monocytes (350,000 cells/mL) were stimulated with 1 μg/mL EV. After 24 h (48 h for fibroblasts), glucose uptake and lactate production were measured using the D‐Glucose Enzymatic Assay Kit (Biosentec, 010) and the L‐Lactic Acid Enzymatic Assay Kit (Biosentec, 022) according to the manufacturer's protocol. For lactate measurements, media was first deproteinized by filtration using Microcon‐10 kDa Centrifugal Filter Units (Millipore, MRCPRT010). Alternatively, EV mediated changes in glucose consumption were determined after endocytosis inhibition (Wortmannin, Alomone labs, SL‐2052) or after inhibiting mTOR (rapamycin, MedChemExpress, HY‐10219). Cells were pre‐incubated with 1 μM Wortmannin or 55 nM rapamycin for 30 min, after which the medium was replaced with medium containing 1 μg/mL EV and 1 μM Wortmannin or 55 nM rapamycin.

### 2.8 Glucose tracing

Endothelial cells were seeded at a density of 28,000 cells/cm^2^ and allowed to attach for 6 h, after which the cells were washed with PBS and medium was changed to glucose‐free RPMI (Sigma—R1383), supplemented with 2 g/L ^13^C_6_‐labelled glucose, 10% dialyzed FBS (Gibco, A3382001) and 1 μg/mL EV for 48 h. Afterwards, cells were washed in 0,9% NaCl and extraction of metabolites from cells and medium was performed by overnight incubation at −80°C with extraction buffer containing 80% methanol and 2 μM d27 myristic acid. Next, samples were analyzed via mass spectrometry as described previously (Kampen et al., 2019). Metabolite abundances in cells and uptake/secretion rates were normalized to protein content and total amount of cells, respectively.

### 2.9 De novo protein synthesis

Endothelial cells were seeded at a density of 28,000 cells/cm^2^ and allowed to attach for 6 h. Subsequently, cells were stimulated with 1 μg/mL EV for 44 h, after which cells were washed with PBS and medium was replaced by methionine free RPMI (Gibco, A14517‐01) supplemented with 10% FBS to deplete methionine reserves for 1 h. Afterwards, medium was replaced by methionine free RPMI supplemented with 10% FBS and 50 μM L‐Azidohomoalanine (Invitrogen, C10102). After 3 h cells were lysed in lysis buffer (50 mM Tris‐HCl, 1% SDS and protease inhibitors (Roche, 11873580001)) for 30 min on ice. After sonication, the click‐it reaction was performed on equal amounts of protein using the Click‐iT Protein reaction buffer kit (Invitrogen, C10276) and biotin (Invitrogen, B10185) according to the manufacturer's protocol. Biotin was visualized using streptavidin‐HRP labelling and chemiluminescence capture (Amersham ECL Prime Western Blotting Detection Reagent (Cytiva, RPN2232)) and imaged using the Azure C600 imaging system (Azure Biosystems). Lane intensity was quantified using ImageJ software. Total, small, intermediate and large proteins were analysed based on empirical observations.

### 2.10 Chicken chorioallantoic membrane assay

To evaluate EV induced angiogenesis, we performed a CAM assay as previously described (Bronckaers et al., 2013). In short, fertilized chicken eggs (*gallus gallus*) were incubated at 37°C at constant humidity for 3 days. After 3 days of embryonic development (E3), 3 mL albumen was removed to detach the egg shell from the developing chorioallantoic membrane (CAM). Subsequently a 1 cm^2^ window was created in the shell, exposing the CAM. The window was covered with cellophane tape and the eggs were returned to the incubator. At E9, 25 μL EV (3.8 × 10^9^ EV) were mixed with 10 μL growth factor reduced Matrigel (BD, Franklin lakes, NJ) and allowed to solidify at 37°C for 2 h. Next, the solidified Matrigel droplets were applied onto the CAM, Matrigel droplets without EV (25uL PBS + 10uL Matrigel) were used as negative controls. Afterwards, the windows in the egg shell were covered and the eggs were incubated for 72 h at 37°C. At E12 the CAM was removed and blood vessel formation was evaluated. Pictures were taken using a digital camera (Nikon DN100 Digital Net Camera). All vessels intersecting two concentric circles (with a radius of 6 and 8 mm) digitally positioned around the Matrigel droplets were counted double blind and independently by three investigators.

### 2.11 Subcellular fractionation and detection of EV derived proteins

Endothelial cells were exposed to L‐AHA‐labelled EV (20 μg/mL) for 24 h. Afterwards, subcellular fractionation was performed via density gradient centrifugation. Cells were harvested in buffer containing 0.25 M sucrose, 1 mM EDTA, 10 mM acetic acid, protease inhibitors (Roche, 11873580001), pH 7.4, and incubated on ice for 20 min. Afterwards, cells were disrupted by passing them through a 22G needle and a clear homogenate was generated by two consecutive centrifugations at 200 g for 5 min. The supernatant (1 mL) was loaded on top of a discontinuous OptiPrep (Serumwerk Bernburg, 1893) gradient (1 mL each of 70, 65, 60, 50, 45, 40, 35, 30, 20% (vol/vol in PBS) solutions) and submitted to ultracentrifugation at 268,000 g for 16 h (Optima XPN‐90 Ultracentrifuge, SW41 rotor, Beckman Coulter). Ten fractions (1 mL) were collected, sonicated and either mixed with (non)‐reducing Laemli (for immunoblotting) or precipitated and processed for the click‐it reaction as described above.

### 2.12 RT‐qPCR

RNA was isolated using the NucleoSpin kit (Machery‐Nagel, 740955.50) and converted into cDNA using the iScript cDNA Synthesis Kit (Biorad, 1708891). Gene expression (Table S1) was analysed using the SensiMix SYBR® Low‐ROX Kit (meridian bioscience, QT625‐05) in the CFX connect Real‐Time System (Biorad) and normalised to RPL13A expression.

### 2.13 Western blot

For western blot on EV derived proteins, 0,9 μg of EV were pelleted by ultracentrifugation at 100,000 g for 60 min at 4°C (Optima XPN‐90 Ultracentrifuge, SW 41Ti rotor, Beckman Coulter) and lysed in RIPA buffer supplemented with either non‐reducing sample buffer (for immune probing of tetraspanins, 3:1, 0,5 M Tris‐HCl, 30% glycerol, 10% SDS, 200 μM bromophenol blue) or reducing sample buffer (all other antigens, 3:1, non‐reducing sample buffer + 5% β‐mercaptoethanol). Protein lysates were denatured for 10 min at 70°C (after click‐it reaction) or for 5 min at 96°C (all other lysates), separated by SDS‐PAGE and transferred to a PVDF membrane. Membranes were blocked in 5% casein in PBS‐T (0,2% Tween‐20 in PBS, for immune probing of EV markers), 5% BSA in PBS‐T (for immune probing of click‐it samples) or 5% BSA in TBS‐T (0,2% Tween‐20 in TBS, for immune probing of all other antigens) for 90 min. Next, transferred proteins were probed using streptavidin‐HRP (1/1000, R&D systems, 890803, for click‐it samples) or antibodies raised against CD63 (0,5 μg/mL, BD Pharmingen, 556019), CD81 (0,5 μg/mL, BD Pharmingen, 555675), GM130 (0,25 μg/mL, BD Biosciences, 610823), TSG101 (0,2 μg/mL, Santa Cruz Biotechnology, SC‐7964), Syntenin‐1 (0,5 μg/mL, Abcam, ab133267), P‐mTOR (Ser2448) (0,43 μg/mL, Cell Signaling, 5536S), P‐RPS6K (Thr389) (1,49 μg/mL, Cell Signaling, 9206S), RPS6K (0,042 μg/mL, Cell Signaling, 9202S), 4E‐BP1 (0,169 μg/mL, Cell Signaling, 9644S), P‐4E‐BP1 (Ser65) (0,08 μg/mL, Cell Signaling, 9451S), P‐AMPKα (Thr172) (0,027 μg/mL, Cell Signaling, 2535S), Rab5 (0,022 μg/mL, Cell Signaling, 46449S), Cathepsin L (0,043 μg/mL, Cell Signaling, 71298S), LAMP1 (0,6 μg/mL, Abcam, ab24170), AKT (0,035 μg/mL, Cell Signaling, 4691S), EIF4E (1/1000, Transduction Laboratories, E27620), ATP synthase (1,5 μg/mL, Abcam, ab110413), Histone 3 (0,009 μg/mL, Cell Signaling, 9715S), β‐actin (1/10.000, Clone 4, MP Biomedicals, 0869100‐CF) overnight at 4°C. After incubation with their respective HRP labelled secondary antibodies (Anti‐mouse, 0,184 μg/mL, Cell Signaling, 7076S; Anti‐rabbit, 0,06 μg/mL, Cell Signaling, 7074S) for 90 min, bands were visualized using Amersham ECL Prime Western Blotting Detection Reagent (Cytiva, RPN2232) and imaged using the Azure C600 imaging system (Azure Biosystems). Band intensity was quantified using ImageJ software.

### 2.14 Mass spectrometry of EV associated proteins

EV proteins were precipitated by overnight incubation at −20°C with 1.5 mL acetone containing 10% (v/v) trichloroacetic acid (TCA; Sigma–Aldrich) and 20 mM 1,4‐dithiothreitol (DTT; Sigma–Aldrich) followed by centrifugation for 10 min at 16,000 g and 4°C. After washing with ice cold acetone and repeated centrifugation, the protein pellets were resuspended in 30 μL 50 mM ammonium bicarbonate (ABC; Sigma–Aldrich) containing 5M urea. To evaluate membrane protein expression after EV stimulation, membranes were isolated using the Mem‐PER plus kit (Thermo Scientific, 89842Y), according to the manufacturer's protocol. Protein concentration was determined using the Pierce BCA Protein Assay Kit (thermos Fisher, 23227) according to the manufacturer's instructions.

To 5 μg of EV‐protein, or 30 μg of membrane proteins, DTT (20 mM) was added and incubated at RT for 45 min. Iodoacetamide (40 mM) was then added to alkylate for 45 min at RT until quenching by a second addition of DTT. A mixture of trypsin/lysC (2 μg) was added and digestion was performed at 37°C for 2 h. The mixture was diluted with ABC without urea and incubation was continued at 37°C for 18 h. The digestion mix was briefly centrifuged and the digest/peptide mixture diluted fourfold for nano‐LC MS/MS analysis. A nanoflow HPLC instrument (Dionex ultimate 300) was coupled in‐line with a Q Exactive (Thermo Scientific) with a nano‐electrospray Flex ion source (Proxeon). Five microliter of the digest/peptide mixture was loaded onto a C18‐reversed phase column (Thermo Scientific Acclaim PepMap C18 column, 75 μm inner diameter × 50 cm, 5 μm particle size). The peptides were separated with a 240 min linear gradient of 4%–45% buffer B (80% acetonitrile and 0,08% formic acid) at a flow rate of 300 nL/min. MS data were acquired using a data‐dependent top10 method, dynamically choosing the most abundant precursor ions from the survey scan (250−1250 *m*/*z*) in positive mode. Survey scans were acquired at a resolution of 70,000. Dynamic exclusion duration was 30 s. Isolation of precursors was performed with a 4.0 *m*/*z* window. Resolution for HCD spectra was set to 17,500 and the Normalized collision energy was 30 eV. The under fill ratio was defined as 1.0%. The instrument was run with peptide recognition mode enabled, but exclusion of singly charged and charge states of more than five. The data were analysed using Sequest HT Proteome Discoverer 2.2 search engine (Thermo Scientific), against the Uniprot database. The false discovery rate (FDR) was set to 0,01 for proteins and peptides, which had to have a minimum length of 6 amino acids. The precursor mass tolerance was set at 10 ppm and the fragment tolerance at 0,2 Da. One miss‐cleavage was tolerated, oxidation of methionine was set as a dynamic modification and carbamidomethylation of cysteines was a fixed modification. Proteins with a score > 10 were considered as being identified with high confidence (adapted from (Benedikter et al., 2017)).

### 2.15 EV track score

All relevant data were submitted to the EV‐TRACK knowledgebase (EV‐TRACKID: EV230033, Average EV‐Metric score 58.33%) (Van Deun et al., 2017).

### 2.16 Statistics

Data were analysed using GraphPad Prism software. Statistical significance was determined using an ANOVA test with Dunnett's multiple comparisons post‐hoc test, unless indicated otherwise. Data were considered statistically significant when *p* < 0,05. All data are presented as mean ± SEM.

## 3 RESULTS

### 3.1 Characterization of cancer cell derived extracellular vesicles

**FIGURE 1:**
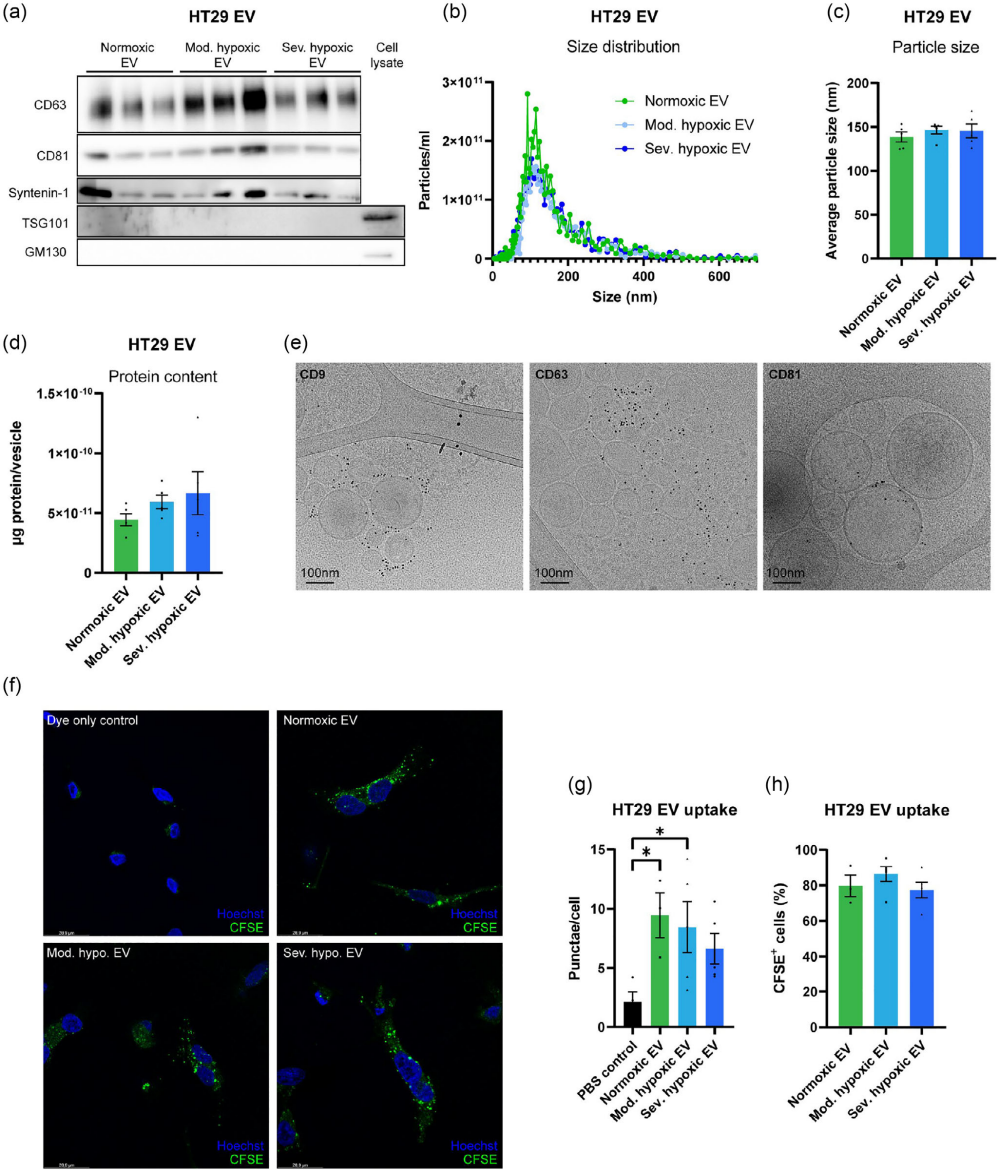
Characterization and uptake of HT29 derived extracellular vesicles. EV derived from HT29 cells exposed to normoxia or moderate/severe hypoxia for 24 h were isolated using size exclusion chromatography and characterized in line with the MISEV2018 guidelines. (a) Immunoblot analysis demonstrates the presence of CD63, CD81 and Syntenin‐1, and the absence of TSG101 and GM130 in HT29 derived EV. *n* = 3. (b) Nanoparticle Tracking Analysis (NTA) demonstrates a typical EV size distribution. *n* = 5, mean. (c) Average particle size was not significantly altered after exposure of HT29 cells to either normoxia or moderate/severe hypoxia. *n* = 5, mean ± SEM. (d) Protein content in vesicles is not significantly altered in EV derived from moderate/severely hypoxic HT29 cells. *n* = 5, mean ± SEM. (e) Transmission electron microscopy of immunogold labelled EV isolated from severely hypoxic cancer cells demonstrates the presence of CD9, CD63 and CD81 on lipid bilayer vesicles. (f) Endothelial cells (HUVEC) exposed to CFSE‐labelled EV demonstrated a punctuated intracellular staining pattern, indicating the uptake of EV. (g) The amount of punctae were quantified using ImageJ software and normalized to the number of cells. Per condition an average of 100 cells were quantified. (h) The percentage of CFSE positive cells was not significantly altered after stimulation with either normoxic or moderate/severely hypoxic EV. Per condition an average of 100 cells were counted. Mean ± SEM, *p < 0,05.

Extracellular vesicles were isolated and characterised in line with the MISEV2023 guidelines to guarantee that observed effects could be attributed to a pure EV isolate rather than co‐isolated contaminants (Welsh et al., 2024). Colorectal adenocarcinoma (HT29), glioblastoma (U87) and breast adenocarcinoma (MDA‐MB‐231) cells were exposed to normoxia (21% O_2_), moderate hypoxia (0,2% O_2_) or severe hypoxia (O_2 _< 0,02%) for 24 h. Immunoblot analysis demonstrates EV markers CD63 and CD81 in HT29, U87 and MDA‐MB‐231 cells and EV (Figure 1a, Figures S1A,B and S2). Furthermore, syntenin‐1 is present in EV from HT29 and U87 cells. Unexpectedly, all EV isolates are negative for EV marker TSG101. The negative EV marker Golgi matrix protein 130 (GM130) was not detected in any of the EV isolates. Nanoparticle tracking analysis (NTA) of EV preparations reveals a typical particle size distribution (Figure 1b, Figure S1C,DS1). Particle size and total protein content is irrespective of producer cell oxygenation status (Figure 1c, d, Figure S1E–H). Cryo‐transmission electron microscopy analysis of (immunogold labelled) EV confirms the isolation of vesicles with lipid bilayers positive for EV markers CD9, CD63 and CD81 (Figure 1e, Figure S3). In summary, these results confirm the isolation of extracellular vesicles from HT29, U87 and MDA‐MB‐231 cells with typical morphology, size distribution and marker expression.

### 3.2 Uptake of colorectal cancer cell derived EV by endothelial cells

EV can elicit functional effects on recipient cells in various ways. Firstly, ligands present on EV can activate receptors on the cellular membrane (van Niel et al., 2018). In addition, EV can be internalized by, or fuse with, recipient cells via multiple pathways and subsequently release their molecular cargo into the target cell (van Niel et al., 2018). To evaluate EV uptake by endothelial cells, cancer cell derived EV were labelled with Carboxyfluoresceïne Succinimidyl Ester (CFSE), incubated with endothelial cells and assessed by confocal microscopy. Endothelial cells stimulated with HT29 EV demonstrate a punctuated intracellular staining pattern, indicating the uptake of CFSE labelled EV (Figure 1f). No differences in uptake of EV derived from either normoxic or moderate/severe hypoxic cells are observed (Figure 1g, h). Flow cytometry confirms the uptake of HT29 EV by endothelial cells, indicated by an increase in the percentage of CFSE^+^ endothelial cells (Figure S4A, B). No change in mean fluorescence intensity between normoxic and moderate/severe hypoxic EV stimulated cells is observed (Figure S4A–C). In contrast, stimulation of endothelial cells with labelled U87 and MDA‐MB‐231 derived EV does not significantly alter the percentage of CFSE^+^ cells (Figure S4D‐E), suggesting differences in kinetics or that ligand binding, rather than uptake, is their main mode of action.

### 3.3 Cancer cell‐derived EV contain glycolytic enzymes, transfer cargo to respective organelles and increase glucose metabolism in recipient cells

**FIGURE 2:**
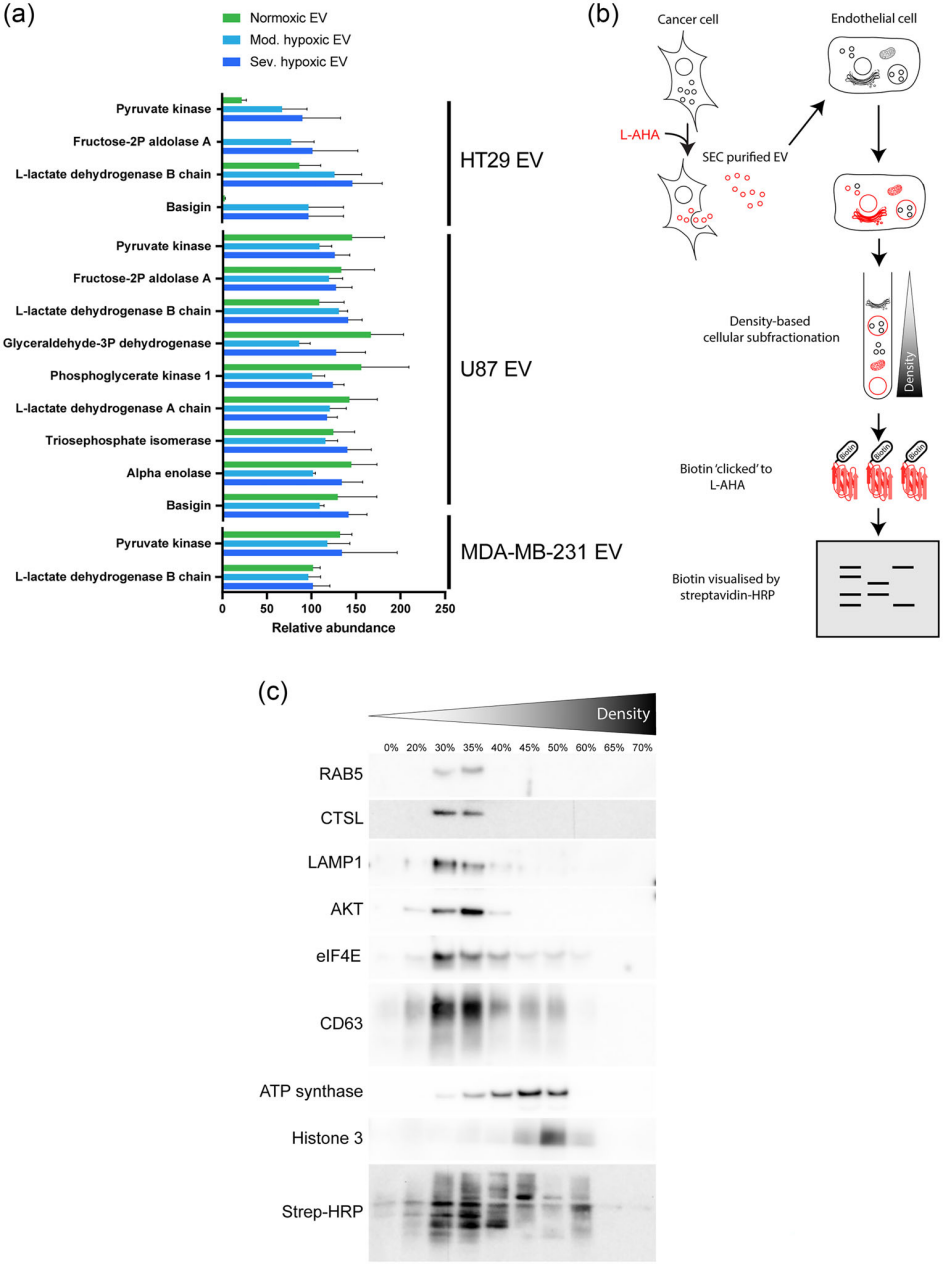
Cancer EV transport glycolytic enzymes to recipient endothelial cells. (a) Mass spectrometry of HT29, U87 and MDA‐MB‐231 cell derived EV reveals the presence of various glycolytic enzymes in cancer cell derived EV. The secretion of these enzymes in HT29 derived EV is depending on producer cell oxygenation status. *n* = 3, mean ± SEM. (b) Schematic overview of EV‐cargo tracing. Cancer cells were cultured with the methionine analog, L‐AHA, for 24 h followed by EV purification using SEC. Next, recipient endothelial cells (HUVEC) were exposed to L‐AHA labelled EV for 24 h. Subcellular fractionation was performed via density gradient centrifugation. Biotin was `clicked` to EV delivered, L‐AHA labelled proteins in the different cellular subfractions and visualized using streptavidin‐HRP. (c) EV derived proteins were detected in all the different cellular subfractions, indicating that after endocytosis, EV derived proteins escape the endolysosomal pathway and reach different intracellular compartments. RAB5 = Ras‐related protein Rab‐5A (early endosomes), CTSL = Cathepsin L (lysosomes), LAMP1 = Lysosome‐associated membrane glycoprotein 1 (lysosomes), AKT = RAC‐alpha serine/threonine‐protein kinase (cytoplasm), EIF4E = Eukaryotic translation initiation factor 4E (cytoplasm/nucleus), ATP synthase = ATP synthase F1 subunit alpha (mitochondria), CD63 (late endosomes), Histone 3 (nucleus).

Deregulation of cellular metabolism has been recognized as one of the hallmarks of cancer (Hanahan & Weinberg, 2011). In contrast to non‐malignant cells, cancer cells prefer aerobic glycolysis over oxidative phosphorylation for the production of ATP (Hanahan & Weinberg, 2011; Vander Heiden et al., 2009). Proteomic analysis of HT29, U87 and MDA‐MB‐231 derived EV demonstrates the presence of various glycolytic enzymes, including pyruvate kinase PKM, L‐lactate dehydrogenase and glyceraldehyde‐3‐phosphate dehydrogenase (Figure 2a, Tables 2–4). Interestingly, HT29 cells increase the secretion of these enzymes via EV during moderate and severe hypoxia. Furthermore, HT29 and U87 derived EV contain Basigin (CD147), which has previously been described to increase glucose uptake and glycolysis in several cancers (Huang et al., 2014; Li et al., 2019; Li et al., 2020). To evaluate whether these EV‐associated enzymes reach their respective intracellular compartments in endothelial cells after EV uptake, we next developed an EV‐cargo tracing assay. In short, to distinguish and label proteins delivered via EV, L‐Azidohomoalanine (L‐AHA) replaced methionine in the culture medium of EV producing cells. After subcellular fractionation of recipient cells, click‐it chemistry (biotin) allows determination of delivered protein distribution (Figure 2b). As expected, EV‐derived proteins were detected in fractions associated with early endosomes, lysosomes and late endosomes. Yet evidence for escape from the endosomal pathway is provided by the presence of L‐AHA labelled proteins in cytoplasm, mitochondria, endoplasmatic reticulum and nuclei (Figure 2c). Furthermore, EV‐derived proteins demonstrate a distinct band‐pattern in each of the subcellular fractions, indicating targeting of specific EV cargo to different subcellular compartments (Figure 2c). Taken together, we demonstrate the transfer of proteins from cancer derived EV to endothelial cells, suggesting that cancer derived EV contribute to recipient cell (metabolic) reprogramming.

**FIGURE 3:**
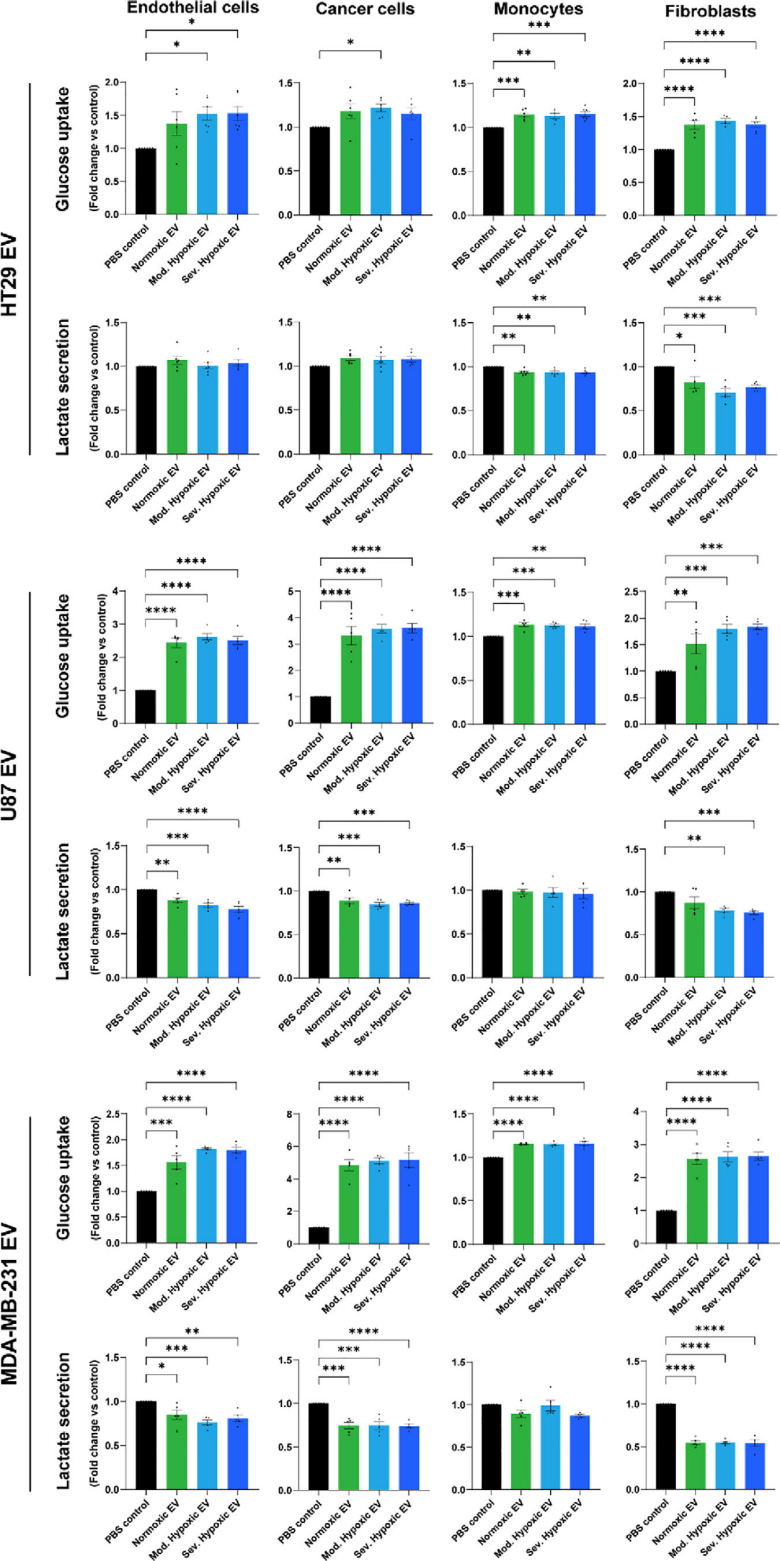
Cancer derived EV stimulate glucose uptake in recipient cells. Glucose uptake and lactate secretion by recipient HUVEC‐ and cancer‐cells, monocytes and fibroblasts were measured after 24 h EV stimulation. Overall, glucose uptake was increased upon stimulation with cancer derived EV. Lactate secretion did not increase after cancer derived EV stimulation. *n* = 6 (HT29 EV), *n* = 5 (U87 EV, MDA‐MB‐231 EV), mean ± SEM. *p < 0,05 **p < 0,01 ***p < 0,001 ****p < 0,0001.

Next, we evaluated the capacity of cancer cell derived EV (HT29, U87 and MDA‐MB‐231) to reprogram glucose metabolism in endothelial‐ and cancer‐ cells, monocytes and fibroblasts. Cancer cell derived EV increase glucose uptake in recipient cells irrespective of EV producing cell oxygenation status (Figure 3, Figure S11). To validate whether the observed metabolic effects of EV on recipient cells are cancer‐specific, rather than general trophic effects, we analysed endothelial glucose uptake after stimulation with EV derived from healthy or non‐angiogenic cells. Glucose consumption was not altered in endothelial cells after stimulation with MRC5 (lung fibroblasts), BJ (foreskin fibroblasts) or U937 (monocytes) derived EV, indicating cancer‐specificity of EV‐mediated metabolic reprograming (Figure S5). Interestingly, the degree of glucose uptake in cancer EV recipient cells depends on both EV producing‐ and recipient cell type. To evaluate whether the differences in glucose consumption observed between endothelial cells stimulated with different EV types is related to mechanistic differences in EV uptake (Figure S4B,D,E), EV mediated changes in glucose consumption were evaluated after pre‐incubation of endothelial cells with endocytosis inhibitor Wortmannin followed by exposure to EV. Inhibiting endocytosis prevents enhanced glucose consumption in endothelial cells after stimulation with HT29 and U87, but not MDA‐MB‐231 EV (Figure S6). These data support the notion that MDA‐MB‐231 EV exhibit their effect on recipient cells via differential mechanisms. The cancer cell secretome contains a plethora of factors known to alter endothelial metabolism and to drive tumour vascularization. We next investigated the contribution of EV to the overall effect of the cancer cell secretome on recipient endothelial cell metabolism by depleting EV. EV depletion from conditioned medium of hypoxic HT29 cells reduces glucose metabolism in recipients cells, underlining the importance of EV in the overall effect of the cancer cell secretome on recipient cell metabolism (Figure S7).

We next evaluated whether the increased glucose uptake after stimulation with cancer derived EV is solely used for aerobic glycolysis, measuring lactate secretion by EV‐recipient cells. Interestingly, the enhanced consumption of glucose upon EV stimulation does not result in elevated lactate secretion. In contrast, dependent on the acceptor cells, EV stimulation results in decreased lactate production (Figure 3). In conclusion, these results demonstrate the metabolic reprogramming of cells within the TME by cancer derived EV, independent of the oxygenation status of the EV producing cells. Overall, cancer derived EV push recipient cells towards increased glycolytic metabolism. The absence of increased lactate secretion in these cells suggests the use of glucose in downstream biosynthetic pathways, for example, for the synthesis of nucleotides or amino acids.

### 3.4 Cancer cell derived EV stimulate amino acid synthesis and uptake in endothelial cells

Next, we performed ^13^C_6_‐glucose tracing to analyse differentially activated metabolic pathways in reprogrammed endothelial cells in more detail. Endothelial cells were stimulated with HT29, U87 and MDA‐MB‐231 derived EV in medium supplemented with ^13^C_6_‐glucose. Conditioned medium and cell lysates were collected after 48 h and (un)labelled metabolites were analysed using mass spectrometry.

**FIGURE 4:**
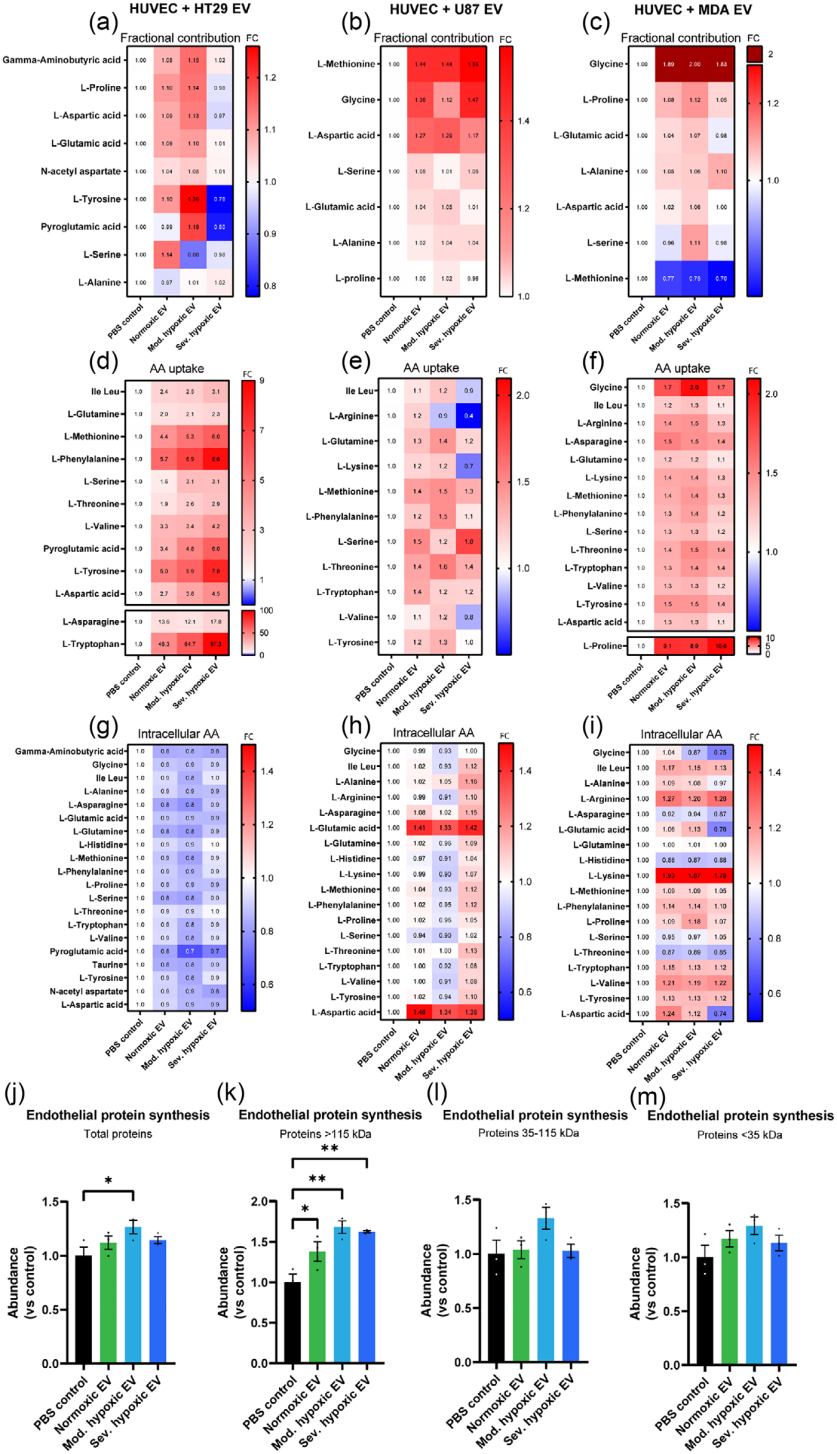
Cancer cell derived EV stimulate amino acid synthesis and uptake in endothelial cells to fuel protein synthesis. Endothelial cells (HUVEC) were stimulated with cancer derived EV in the presence of ^13^C_6_‐glucose. After 48 h, ^13^C‐labelling of downstream metabolites was analysed using mass spectrometry. Upon stimulation with (a) HT29, (b) U87 and (c) MDA‐MB‐231 derived EV, endothelial cells increase the synthesis of AA as measured by an increase in fractional contribution. In addition, uptake of AA by endothelial cells was increased after 48 h of exposure to (d) HT29, (e) U87 and (f) MDA‐MB‐231 derived EV. Intracellular levels of AA in endothelial cells upon stimulation with (g) HT29, (h) U87 and (i) MDA‐MB‐231 derived EV remain relatively stable. *n* = 3, numbers represent fold change (FC) compared to PBS control. *De novo* protein synthesis by endothelial cells upon stimulation with HT29 derived EV was analysed using click‐it chemistry. (j) Endothelial cells increase *de novo* protein synthesis upon HT29 derived EV stimulation. (k–m) The increased synthesis of proteins is most pronounced in proteins > 115 kDa, *n* = 3, mean ± SEM. *p < 0,05 **p < 0,01.

Upon stimulation of endothelial cells with cancer cell‐derived EV, we did not observe relevant changes in the synthesis of metabolites from the pentose phosphate pathway, nucleotides, fatty acids or redox related metabolites (data not shown). However, endothelial cells boost the synthesis of amino acids (AA) upon stimulation with cancer cell derived EV, as measured by an increase in the fractional contribution of ^13^C_6_‐labelled versus unlabelled AA (Figure 4a–c). Despite increased AA synthesis, endothelial expression levels of phosphoserine aminotransferase (PSAT1), D‐3‐phosphoglycerate dehydrogenase (PHGDH), serine hydroxymethyltransferase (SHMT2) or phosphoserine phosphatase (PSPH), enzymes in the serine/glycine synthesis pathway, were not significantly increased upon EV stimulation (Figure S8). Interestingly, the effect of HT29 derived EV on endothelial cell metabolism depends on the oxygenation status of the EV producing cells, with normoxic and moderate hypoxic EV, but not severely hypoxic EV, increasing AA synthesis (Figure 4a). This data suggests differential utilization of glucose by endothelial cell after stimulation with severely hypoxic EV.

In addition to *de novo* synthesis, endothelial cells rely on uptake of AA from the extracellular environment. Uptake of AA by endothelial cells after stimulation with cancer cell‐derived EV was analysed using mass spectrometry. After stimulation with cancer cell‐derived EV, endothelial cells increase the uptake of AA (Figure 4d–f). Only HT29 derived EV modify endothelial AA uptake in an oxygenation status dependent manner (Figure 4d). To evaluate whether increased AA uptake was related to increased expression of AA transporters on the cellular membrane, mass spectrometry analysis of endothelial membrane proteins upon stimulation with HT29 derived EV was performed. Interestingly, expression of transporters was not significantly altered upon EV stimulation (data not shown), suggesting that EV‐induced AA uptake is mediated via a different mechanism. Despite elevated synthesis and uptake of AA, intracellular AA pools are only moderately affected in endothelial cells upon stimulation with cancer cell derived EV (Figure 4g–i). Remarkably, HT29 derived EV decrease endothelial intracellular AA pools irrespective of oxygenation status of the producer cells (Figure 4g). Interestingly, intracellular AA pools after U87 derived EV stimulation depend on EV producing cell oxygenation status (Figure 4h). Intracellular AA levels after MDA‐MB‐231 derived EV stimulation are increased (Figure 4i). In summary, these results indicate that cancer cells, via the secretion of EV, reprogram recipient endothelial cells to increase AA synthesis and uptake. These data suggest that EV induce AA use for protein synthesis in endothelial cells.

### 3.5 HT29 derived EV stimulate *de novo* protein synthesis in endothelial cells to support angiogenesis

*De novo* protein synthesis in endothelial cells upon EV stimulation was evaluated using click‐it chemistry. Endothelial cells were stimulated with cancer derived EV for 44 h, after which they were supplemented with the methionine analogue L‐azidohomoalanine (L‐AHA). After biotin labelling and separation of proteins using SDS‐PAGE, nascent proteins were visualised using streptavidin‐HRP.

**FIGURE 5:**
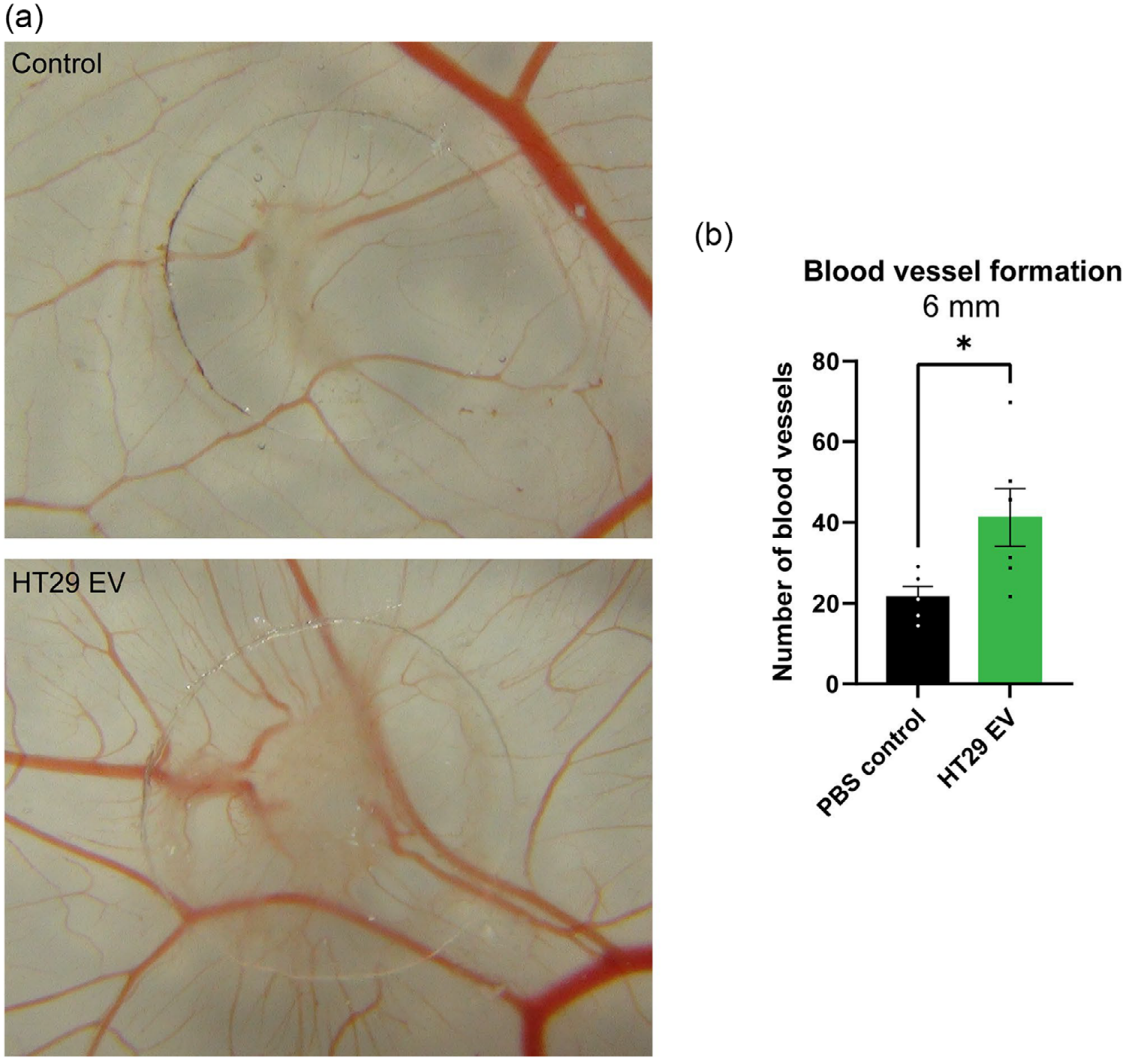
HT29 derived EV stimulate blood vessel formation in an *in ovo* CAM assay. After stimulation with HT29 derived EV for 3 days, vessels intersecting a concentric circle with a radius of 6 mm were counted double blind by three different investigators. (a) Representative pictures of the CAM after treatment with PBS control or HT29 derived EV. (b) Quantification of the amount of blood vessels. HT29 derived EV significantly increase blood vessel formation. *n* = 5 (Control), *n* = 6 (HT29 EV), mean ± SEM. Mann Whitney test, *p < 0,05.

Total levels of newly synthesized proteins in endothelial cells increase upon stimulation with moderate hypoxic HT29 EV (Figure 4j,k, Figure S9). Subdivision of proteins based on size, demonstrates that endothelial cells mainly increase the synthesis of proteins > 115 kDa upon HT29 EV stimulation (Figure 4k). Various endothelial proteins > 115 kDa have been previously demonstrated to play essential roles in blood vessel formation, including Filamin B, Integrin alpha‐5, Filamin‐A, Reticulon‐4, Myoferlin, Clathrin heavy chain 1 and VE‐cadherin (Carmeliet et al., 1999; Del Valle‐Pérez et al., 2010; Fahmy et al., 2016; Li et al., 2020; Stanković et al., 2018; Tung et al., 2013; Xu et al., 2023; Yu et al., 2011; Zhou et al., 2007). As such, increased expression of either of these proteins upon EV stimulation could support angiogenesis. To confirm the angiogenic potential of HT29 derived EV on recipient endothelial cells, we performed an *in ovo* chorioallantoic membrane (CAM) assay. EV significantly increase the formation of new blood vessels after incubation on the CAM for 3 days (Figure 5). Despite enhanced synthesis and uptake of AA, endothelial cells do not alter *de novo* protein synthesis after stimulation with U87 EV (Figure S10A, B), supporting the notion that the effect of EV on recipient cells is cancer type dependent. In summary, we demonstrate that HT29, but not U87, derived EV stimulate *de novo* protein synthesis in endothelial cells to support angiogenesis.

### 3.6 HT29 derived EV activate mTOR and AMPKα in endothelial cells

**FIGURE 6:**
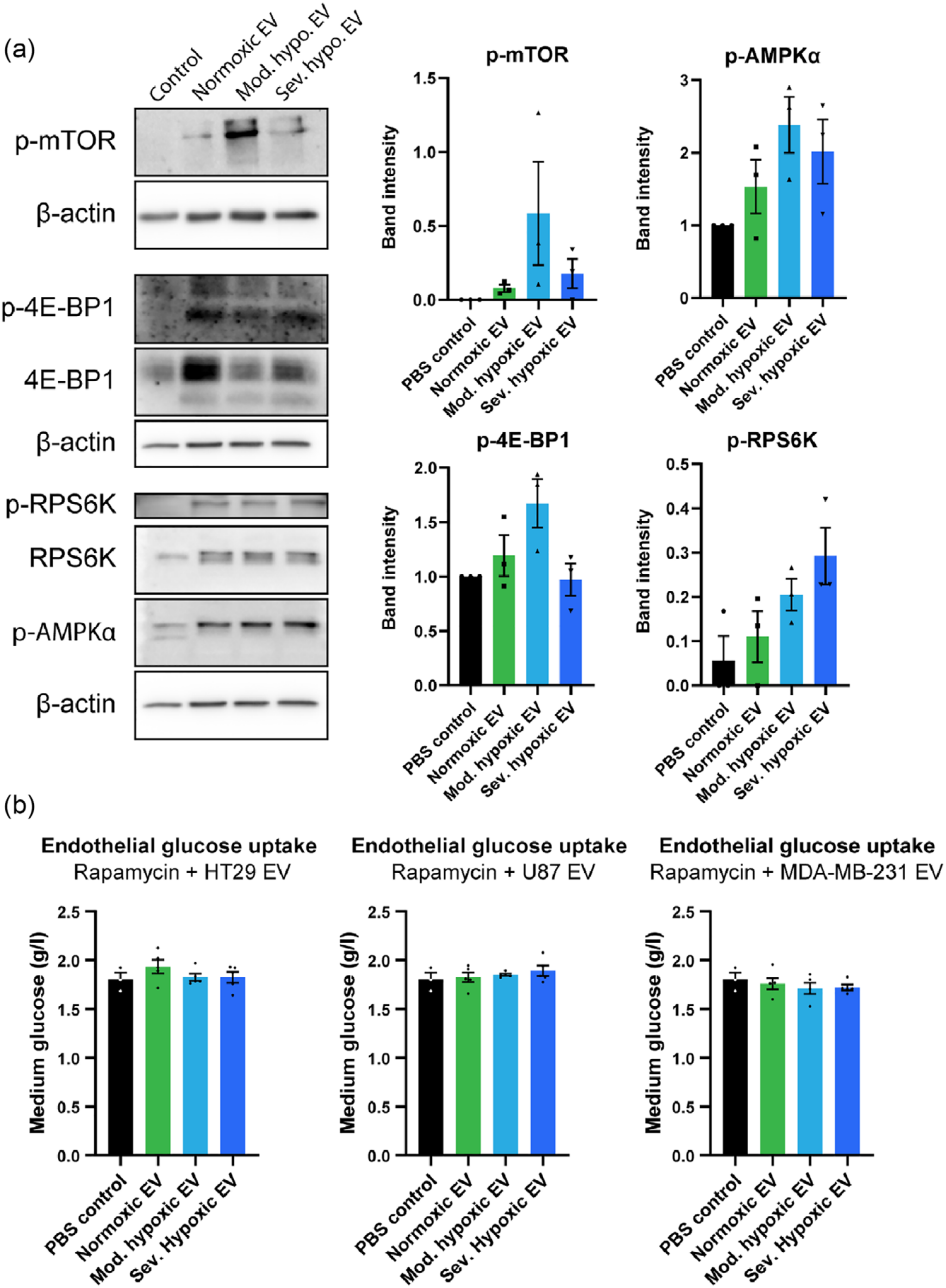
EV induced metabolic reprogramming is mediated via activation of mTOR and AMPKα in recipient endothelial cells. (a) Cells were stimulated with HT29 derived EV for 48 h. Afterwards, phosphorylation of mTOR, AMPKα, 4E‐BP1 and RPS6K was analysed via immunoblotting. Band intensities were normalised to β‐actin (mTOR, AMPKα) or to the total amount of protein of interest (4E‐BP1, RPS6K). *n* = 3, mean ± SEM. (b) Blocking mTOR activation with rapamycin completely abolishes EV induced glucose uptake in recipient endothelial cells (HUVEC). *n* = 5, mean ± SEM.

We next aimed to uncover the pathways involved in the EV mediated increase in glucose consumption and protein synthesis. The AMP‐activated protein kinase α (AMPKα) and mammalian target of rapamycin (mTOR) pathways control glucose metabolism, AA uptake, AA synthesis and protein synthesis (Battaglioni et al., 2022; Herzig & Shaw, 2018; Park et al., 2017; Torrence et al., 2021). We therefore evaluated whether EV increase endothelial glucose consumption and protein synthesis via activation of the mTOR and AMPKα pathways. After 48 h of EV stimulation, endothelial cells were lysed and analysed via immunoblotting. In line with their effect on endothelial glucose consumption, EV increase phosphorylation of AMPKα in recipient cells (Figure 6a). Furthermore, EV induce the phosphorylation of mTOR and its downstream targets 4E‐BP1 and RPS6K (Figure 6a). Similar to the effects of HT29 derived EV on AA uptake, AA synthesis and *de novo* protein synthesis, phosphorylation of mTOR and its downstream targets is most pronounced after stimulation with hypoxic cancer cell derived EV. To evaluate whether EV‐induced mTOR activation was functionally responsible for the effects on endothelial metabolism, endothelial (HUVEC, HMEC) glucose consumption was measured after EV stimulation and mTOR inhibition. Rapamycin addition abolished EV‐induced increases in glucose consumption, confirming that EV‐mediated metabolic reprograming is orchestrated via enhanced activation of mTOR (Figure 6b, Figure S11).

In summary, we demonstrate that HT29 derived EV, via activation of the AMPKα and mTOR pathways, stimulate glucose and AA uptake in endothelial cells to fuel *de novo* protein synthesis to support angiogenesis.

## 4 DISCUSSION

Cancer cells corrupt cells in their surrounding to provide a growth‐promoting environment. Besides the classical means of intercellular communication, this is orchestrated via the release of extracellular vesicles (Meehan & Vella, 2016; Xavier et al., 2020). Metabolic reprogramming of both cancer cells and cells in the TME is one of the hallmarks of cancer and plays an important role in tumour progression (De Bock et al., 2013; Hou et al., 2020; Palsson‐McDermott et al., 2015; Parra‐Bonilla et al., 2010; Penny et al., 2016; Vander Heiden et al., 2009; Yetkin‐Arik et al., 2019). Here, we show that cancer cells, through the secretion of EV, push surrounding cells (malignant and non‐malignant) towards increased glycolysis via phosphorylation of AMPKα and mTOR. Interestingly, EV‐mediated metabolic reprograming is a cancer specific feature, as EV from healthy cells do not affect recipient cell metabolism. Furthermore, we demonstrate that EV‐mediated reprograming is a common phenomenon between vastly different cancer types, which affects various TME resident recipient cells, all of which rely heavily on increased glycolysis to stimulate tumour progression. It should be noted that each of the tested recipient cell lines are intrinsically heterogeneous and the use of single cell lines might not recapitulate the effect of EV on each individual subtype. Using ^13^C_6_‐glucose tracing we show that HT29 EV induced glucose consumption in endothelial cells fuels elevated AA and protein synthesis to support angiogenesis. This study represents the first detailed description of EV‐mediated metabolic reprogramming in endothelial cells and provides mechanistic insights into how cancer derived EV contribute to the generation of a growth stimulating microenvironment. In addition, we show that next to soluble growth factors, EV comprise an important functional part in the whole cancer secretome. Furthermore, we demonstrate that EV‐mediated metabolic reprogramming is not exclusive to endothelial cells, but occurs in different TME‐resident cells including fibroblasts and monocytes, each of which contributes to tumour progression in its own way and warrants further investigation to fully understand the mechanisms underlying EV‐mediated reprogramming of the TME. Lastly, as EV‐mediated metabolic reprograming and induction of angiogenesis functions in parallel to the classical angiogenic pathways (e.g., via VEGF), it potentially provides a resistance mechanism to current anti‐angiogenic therapies such as Avastin, underlining the clinical relevance of our findings.

Despite their involvement in many of the hallmarks of cancer, knowledge about the role of EV in the metabolic reprogramming of the TME remains scarce to date (Fridman et al., 2022; Meehan & Vella, 2016; Xavier et al., 2020). In line with previous reports, we show that cancer cells secrete glycolytic enzymes via their EV and subsequently stimulate glycolysis in recipient cells (Liu et al., 2021; Wang et al., 2020; Zhang et al., 2017; Zhang et al., 2018). Increased glycolysis in cancer cells is linked to therapy resistance and enhanced migration and invasiveness (Liu et al., 2021; Wang et al., 2020). Glycolysis provides cancer cells with essential building blocks to maintain their high proliferation rate (Vander Heiden et al., 2009). Furthermore, lactate produced during glycolysis stimulates tumour growth via the induction of angiogenesis and has profound effects on the functioning of the immune system (Erra Díaz et al., 2018; Végran et al., 2011). As such, autocrine metabolic reprogramming of glucose metabolism by cancer derived EV can stimulate cancer progression by increasing aggressiveness, inducing angiogenesis and modulating anti‐tumour immune reactions. In addition to their autocrine effects, we demonstrate that cancer derived EV stimulate glucose consumption in monocytes, fibroblasts and endothelial cells.

Increased glycolytic flux in monocytes is linked to their differentiation into macrophages which contribute to tumour progression (Hou et al., 2020). Nuclear translocation of the glycolytic enzyme PKM2 upon EV stimulation induces STAT3 phosphorylation to induce the expression of differentiation associated transcription factors MafB, c‐Maf, and Egr‐1 (Hou et al., 2020). Subsequent polarization of macrophages into either the tumour suppressive M1‐ or tumour supportive M2‐phenotype is associated with a metabolic switch towards either glycolysis or oxidative phosphorylation, respectively (Batista‐Gonzalez et al., 2020; Van den Bossche et al., 2017). However, recent literature states that this metabolic discrimination between phenotypes is too simplistic and suggests a crucial role for glycolysis in M2 polarization (Batista‐Gonzalez et al., 2020; Van den Bossche et al., 2017). Indeed, high glycolytic flux in tumour‐associated macrophages supports their ability to stimulate angiogenesis and metastasis (Penny et al., 2016). In line, in its enzymatically active tetrameric form glycolytic enzyme PKM2 attenuates macrophage M1‐phenotype and skews them to the tumour supportive M2 phenotype (Palsson‐McDermott et al., 2015). Collectively, this data shows how increased glycolysis in monocytes/macrophages upon EV stimulation could attenuate anti‐tumour immune reactions. In cancer‐associated fibroblasts (CAFs), enhanced glycolytic flux is linked to their tumour supportive effects (Yang et al., 2021). This increased glycolytic flux in CAFs supports tumour growth by providing metabolites which are used to fuel the pentose phosphate pathway and nucleotide metabolism in cancer cells (Becker et al., 2020; Sung et al., 2020). Furthermore, this metabolic coupling between CAFs and cancer cells is involved in multidrug resistance (Yu et al., 2017). Endothelial cells increase their glycolytic flux upon angiogenic stimulation, which is essential for the development of new blood vessels as it provides a fast way to produce the ATP and precursors for biomass synthesis needed to proliferate and migrate (Fitzgerald et al., 2018; Parra‐Bonilla et al., 2010; Yu et al., 2017). In addition, glycolysis is important for the formation of endothelial tip cells, whose migratory phenotype guides the formation of new blood vessels (De Bock et al., 2013; Yetkin‐Arik et al., 2019). Previously, we have shown that hypoxic cancer cells stimulate angiogenesis via the secretion of EV (Keulers et al., 2021). Here, we demonstrate that HT29 EV stimulate glucose metabolism in endothelial cells via the activation of mTOR to support increased nascent protein synthesis and stimulate angiogenesis. As such, EV induced metabolic reprograming and angiogenesis could potentially act in parallel to the classic angiogenic pathways (e.g., VEGF) and provide a resistance mechanism to current anti‐angiogenic therapies such as Bevacizumab/Avastin (Garcia et al., 2020; Itatani et al., 2018; Tanne, 2011). Surprisingly, increased endothelial AA uptake was not mediated via increased expression of AA transporters on the endothelial membrane. However, cellular AA uptake is affected by various other factors, including the activity of the AA transporters, energy availability and concentration gradients of AA and ions (Hyde et al., 2003; Poncet & Taylor, 2013). In contrast, increased endothelial glucose consumption after stimulation with U87 derived EV did not fuel increased protein synthesis. One explanation might be the use of glucose for glycogen synthesis. Indeed, we observed increased ^13^C‐labelling and intracellular levels of uridine diphosphate hexose, a glycogen precursor, in endothelial cells after hypoxic U87 EV stimulation (data not shown). Knowledge about the role of glycogen metabolism in endothelial cells remains relatively scarce to date. However, endothelial cells have been suggested to use glycogen as a back‐up energy source for migration into glucose‐low regions (Bierhansl et al., 2017). Taken together, EV mediated metabolic reprograming of cells within the TME can support tumour growth by providing nutrients, suppressing immune reactions and inducing blood vessel formation. Indeed, our experiments provide evidence that cancer cell derived EV aid in endothelial cell reprogramming. However, it should be noted that in situ endothelial cells are exposed to continuous gradients of EV, rather than single doses used in our in vitro experiments. In addition, due to the heterogeneous character of tumours, the ratio of cancer cells versus endothelial cells differs between cancer regions (Smits et al., 2014). On average, endothelial cells comprise between <1% and 7% of the total tumour and cancer cells up to 50% (Blanchard et al., 2022; Zhang et al., 2022). In our studies, we have used a dose (1 μg/mL) equivalent to 130 cancer cells per endothelial cell.

The alterations in EV subpopulations and their molecular content upon exposure to hypoxia suggest that these EV elicit different effects in recipient cells compared to normoxic cell derived EV (Keulers et al., 2021; Kucharzewska et al., 2013; Walbrecq et al., 2020; Walbrecq et al., 2020). Indeed hypoxic, rather than normoxic, EV have been previously linked to the suppression of anti‐tumour immune reactions and the transfer of hypoxia tolerance in tumours (Beaumont et al., 2022; Zonneveld et al., 2019). Here, we show that the effect of cancer derived EV on recipient cell glucose metabolism is primarily determined by the EV producing cancer type, rather than oxygenation status. Whereas U87 and MDA‐MB‐231 derived EV elicit the biggest increase in endothelial glucose uptake, HT29 derived EV induce a bigger increase in AA synthesis, AA uptake and protein synthesis. These cancer type dependent differences in EV mediated effects may be explained by differences in the secretion of EV subpopulations, EV uptake by recipient cells, EV content, EV concentration and differential targeting of EV to recipient cells by integrins (Hoshino et al., 2015; Ludwig et al., 2018; Tabak et al., 2018). In line, we demonstrate differences in uptake of HT29, U87 and MDA‐MB‐231 EV by endothelial cells. Additionally, blocking EV uptake via endocytosis inhibition ablates HT29 and U87, but not MDA‐MB‐231, EV induced glucose consumption, supporting the notion that cancer type dependent differences could be attributed to differential uptake mechanisms. Rather than imposing a black/white effect on recipient cell phenotype, hypoxia fine‐tunes the effect of EV on recipient cells. HT29 derived EV increase glucose consumption in endothelial cells irrespective of producing cell oxygenation status. However, they only increase endothelial AA synthesis when parental cells are exposed to either normoxia or moderate hypoxia, but not severe hypoxia. Similarly, HT29 derived EV increase AA uptake and mTOR activation by endothelial cells, which is more pronounced with decreasing oxygenation of the producer cells. It should be noted that in our study, ambient oxygen concentrations were used to represent normoxia, whereas physiological oxygen concentrations in tissues are significantly lower, ranging from 1% to 10% oxygen (Stuart et al., 2018). However, due to the low diffusion rate of oxygen through culture medium and cellular respiration, pericellular oxygen tension is significantly lower, ranging from 1% to 16% O_2_ depending on confluency and metabolic activity (Pettersen et al., 2005). Therefore, even though cells are cultured in high ambient oxygen concentrations, obtained results are relevant for studying effects in tissue normoxia. In conclusion, we provide a thorough description of the cancer EV induced metabolic changes in the TME and its implications for angiogenesis and as such provide novel, valuable insights into the role of EV in cancer progression.

## AUTHOR CONTRIBUTIONS

**Joël E. J. Beaumont**: Conceptualization; data curation; formal analysis; investigation; methodology; project administration; validation; visualization; writing—original draft; writing—review and editing. **Lydie M. O. Barbeau**: Data curation; formal analysis; methodology; writing—review and editing. **Jinzhe Ju**: Data curation; formal analysis; investigation; writing—review and editing. **Kim G. Savelkouls**: Data curation; formal analysis; investigation; methodology; validation; writing—review and editing. **Freek G. Bouwman**: Data curation; formal analysis; writing—review and editing. **Marijke I. Zonneveld**: Conceptualization; funding acquisition; methodology; supervision; writing—review and editing. **Annelies Bronckaers**: Data curation; formal analysis; investigation; writing—review and editing. **Kim R. Kampen**: Data curation; methodology; writing—review and editing. **Tom G. H. Keulers**: Conceptualization; formal analysis; funding acquisition; investigation; methodology; project administration; supervision; validation; visualization; writing—original draft; writing—review and editing; **Kasper M. A. Rouschop**. Conceptualization; formal analysis; funding acquisition; investigation; methodology; project administration; supervision; validation; visualization; writing—original draft; writing‐review and editing.

## CONFLICT OF INTEREST STATEMENT

The authors declare no conflicts of interest.

## Supporting information

Supporting Information

Supporting Information
