# Supplementary material for: Cancer EV stimulate endothelial glycolysis to fuel protein synthesis via mTOR and AMPKα activation
Source: J Extracell Vesicles. 2024 Jul 13;13(7):e12449. doi: 10.1002/jev2.12449 (PMC11245686; doi:10.1002/jev2.12449)
Supplement: Supplementary file 1 — Supporting Information [file JEV2-13-e12449-s001.docx]

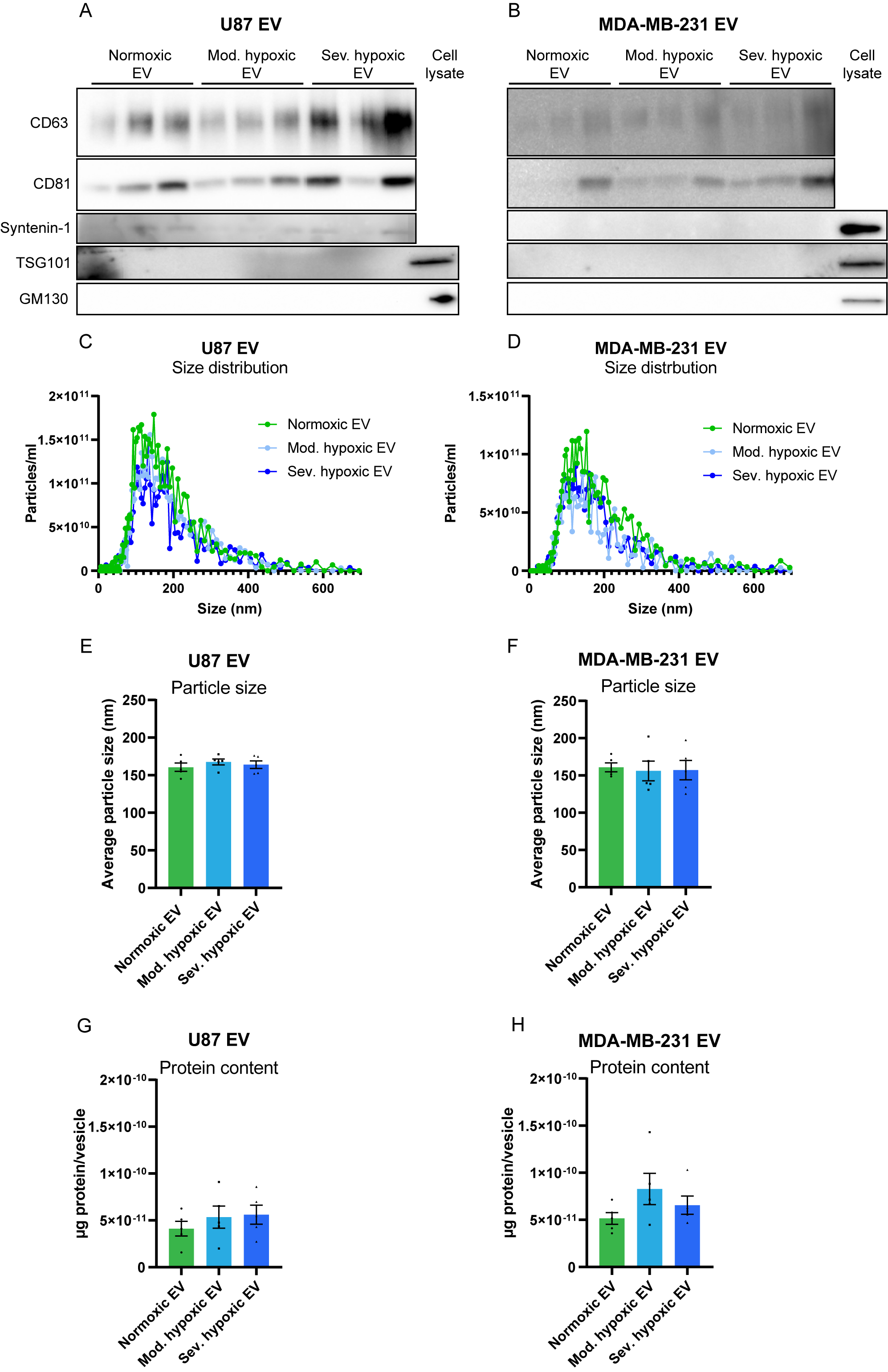


***Supplementary Figure 1: Characterization of U87 and MDA-MB-231 derived extracellular vesicles.*** *EV derived from U87 and MDA-MB-231 cells exposed to normoxia or moderate/severe hypoxia for 24 hours were isolated using size exclusion chromatography and characterized in line with the MISEV2018 guidelines. A,B) Immunoblot analysis demonstrates the presence of CD63, CD81 and Syntenin-1 in U87 derived EV. MDA-MB-231 derived EV are positive for CD63 and CD81. TSG101 and GM130 were not detected in either U87 or MDA-MB-231 derived EV, n=3. C,D) Nanoparticle Tracking Analysis (NTA) demonstrates a typical EV size distribution. n=5, mean. E,F) Average particle size was not significantly altered after exposure of cancer cells to either normoxia or moderate/severe hypoxia, n=5, mean ± SEM. G,H) Protein content in vesicles is not significantly altered in EV derived from moderate/severely hypoxic cancer cells, n=5, mean ± SEM.*

***
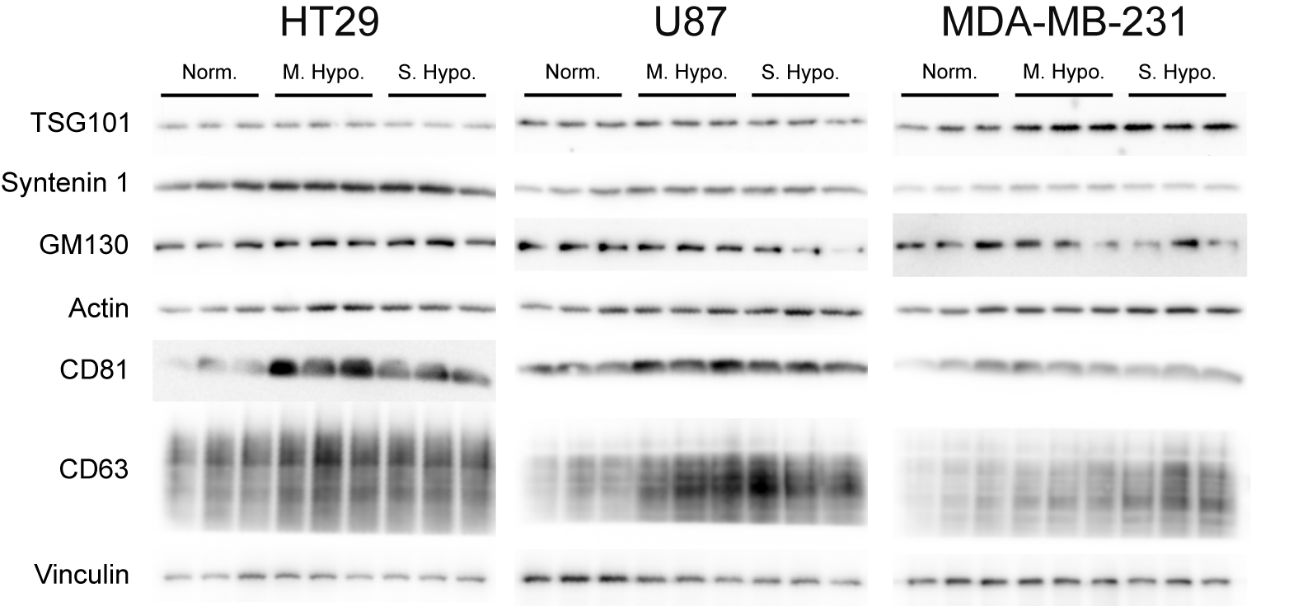
***

***Supplementary Figure 2: Expression of EV markers in cancer cells upon exposure to normoxia or moderate/severe hypoxia.*** *HT29, U87 and MDA-MB-231 cells were exposed to normoxia or moderate/severe hypoxia for 24 hours, after which they were analysed for TSG101, Syntenin1, GM130, CD81 and CD63 expression via immunoblotting. N=3*

***
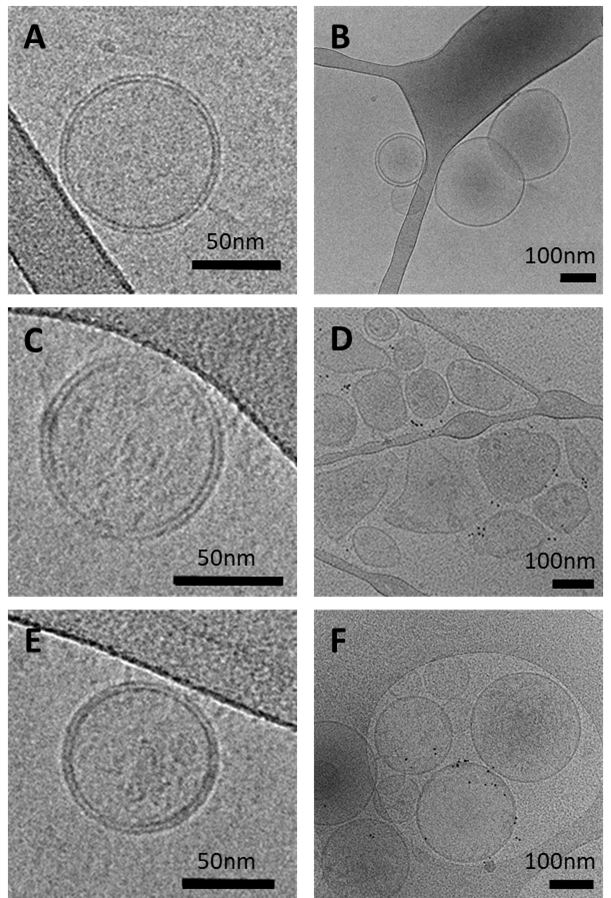
***

***Supplementary Figure 3: Cryo-TEM of HT29 derived EV.*** *Close-up (A, C, E) and overview (B, D, F) EM pictures of HT29 derived EV after exposure of EV producing cells to normoxia (A, B), moderate hypoxia (C, D) or severe hypoxia (E, F).*


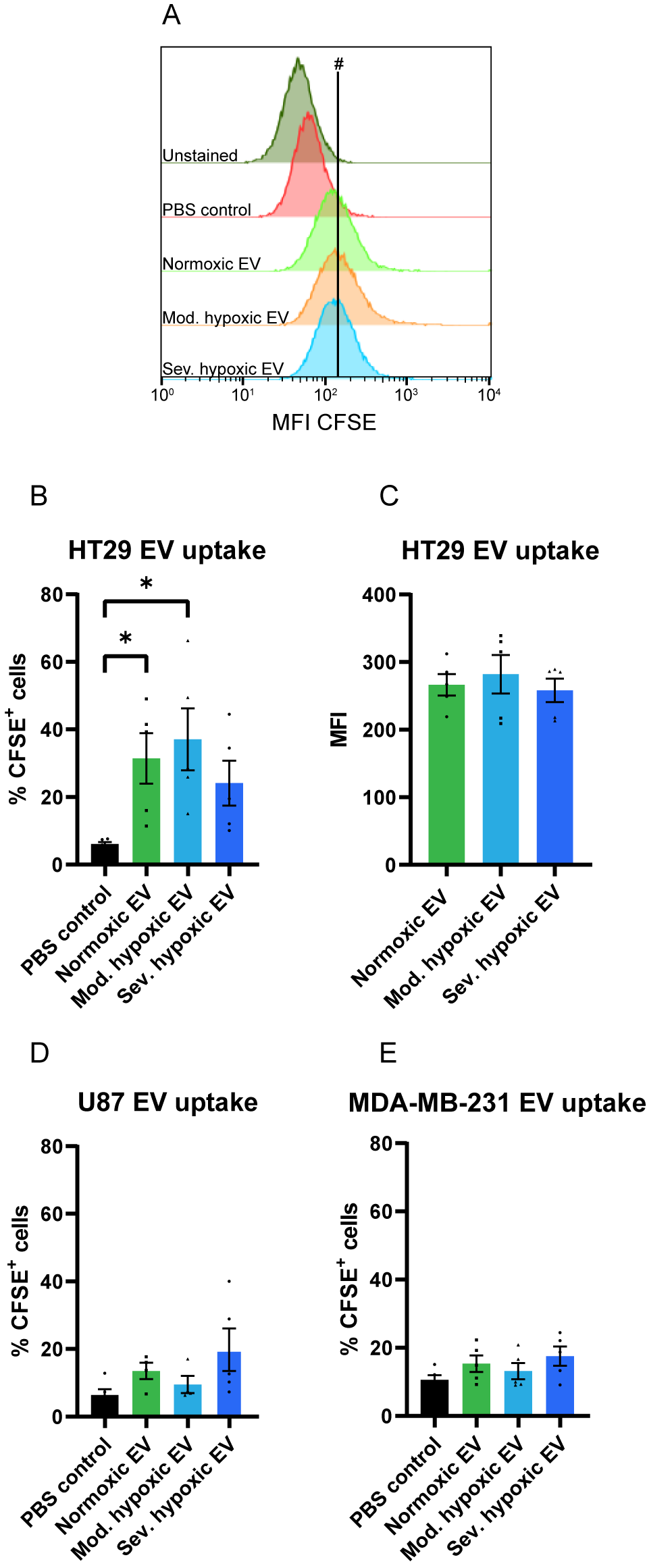


***Supplementary Figure 4: Cancer derived EV uptake by endothelial cells.*** *EV were stained using CFSE and uptake of EV by endothelial cells (HUVEC) was analysed after 6 hours using flow cytometry. A) Fluorescence intensity of endothelial cells, either untreated, stimulated with dye only control, or stained HT29 derived EV. #= cut-off between CFSE-negative and –positive cells based on unstained control B) Percentage of CFSE-positive cells after stimulation with stained HT29 derived EV, n=5, mean ± SEM. C) Mean fluorescence intensity (MFI) of the CFSE-positive endothelial cell population after HT29 EV stimulation, n=5, mean ± SEM. D,E) Percentage of CFSE-positive cells after stimulation with stained U87/MDA-MB-231 derived EV. n=5, mean* *± SEM. *P<0,05*


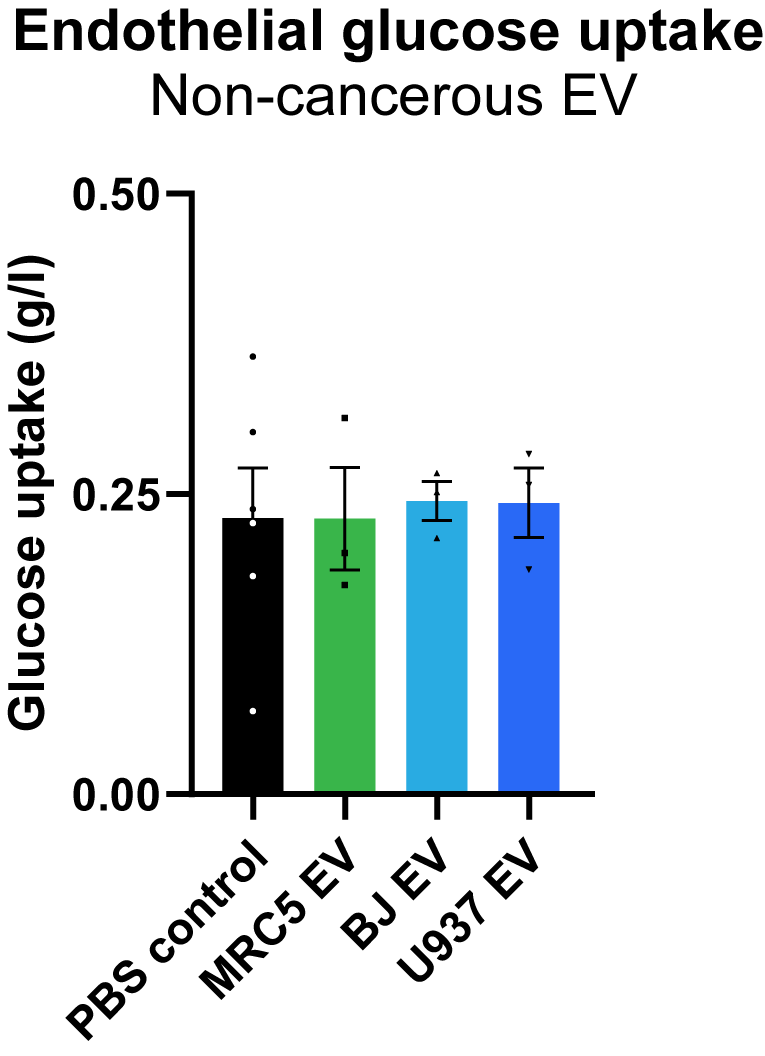


***Supplementary Figure 5: Non-angiogenic cell line derived EV do not induce endothelial metabolic reprograming.*** *Endothelial (HUVEC) glucose consumption was measured after 24 hours stimulation with MRC5 (lung fibroblasts), BJ (foreskin fibroblasts) or U937 (monocytes) derived EV. Glucose consumption was not significantly altered after EV stimulation. Mean ± SEM*


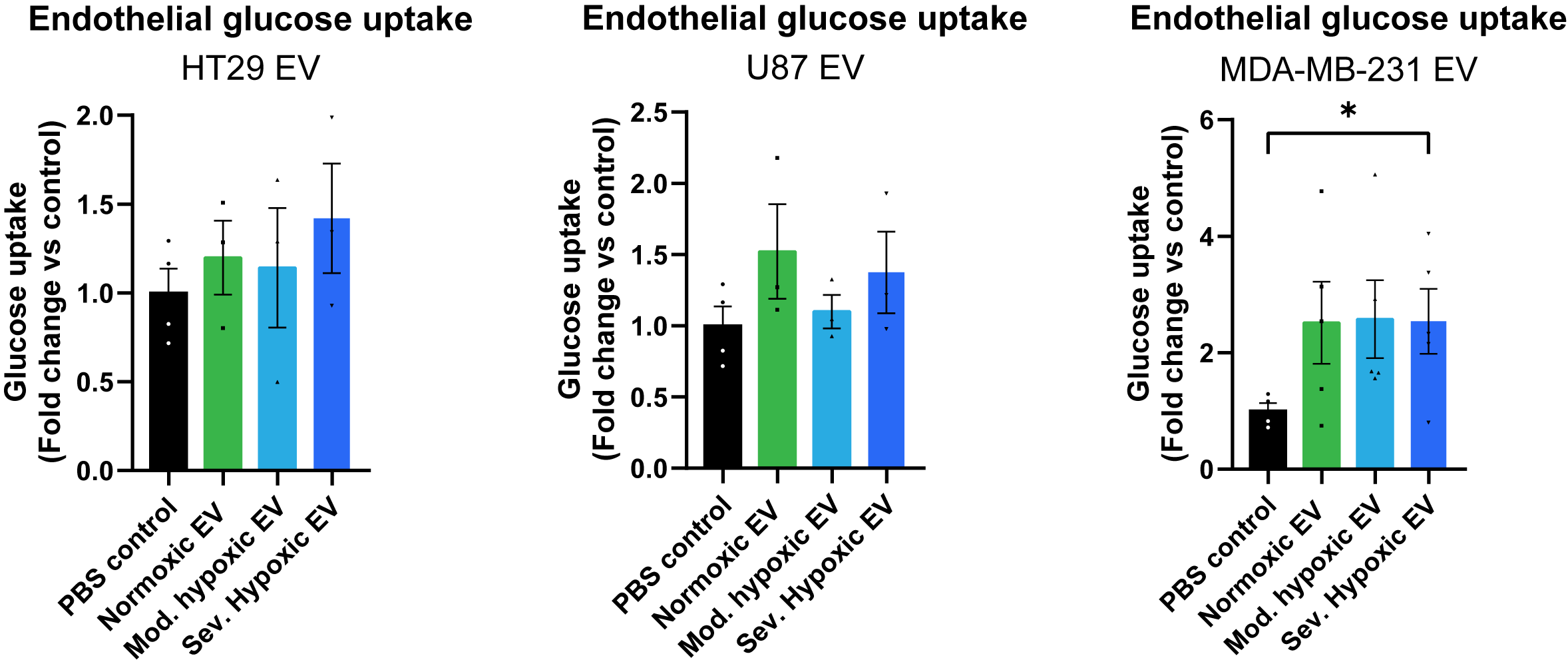


***Supplementary Figure 6: Blocking EV uptake inhibits HT29 and U87 EV induced glucose uptake.*** *Endothelial (HUVEC) endocytosis was blocked by pre-incubation with Wortmannin. Glucose consumption was evaluated after 24 hours stimulation with HT29 (n=3), U87 (n=3) or MDA-MB-231 (n=5) derived EV. Blocking EV uptake inhibits HT29 and U87, but not MDA-MB-231 induced glucose uptake. Unpaired t test, mean ± SEM, *P<0,05*

***
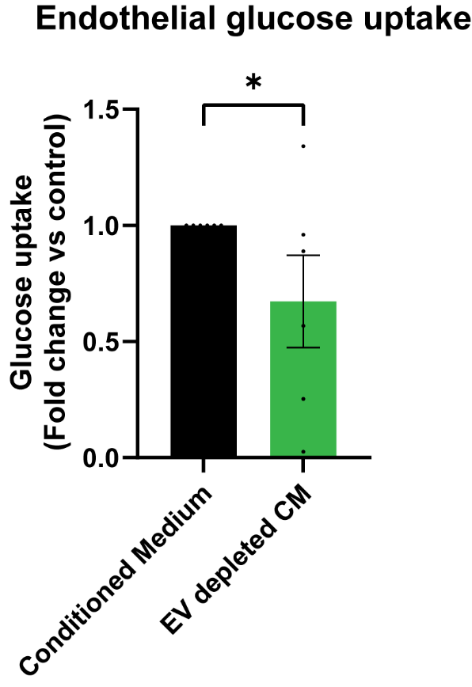
***

***Supplementary Figure 7: EV comprise an important role in the metabolic effects of the cancer secretome.*** *To evaluate the contribution of EV to the metabolic effects of the cancer secretome, endothelial cells (HUVEC) were cultured in (EV depleted) hypoxic HT29 CM. Glucose consumption was measured after 24 hours. Depletion of EV from HT29 CM significantly reduced endothelial glucose consumption. N=6, mean ± SEM, Mann-Whitney test, *P<0,05*


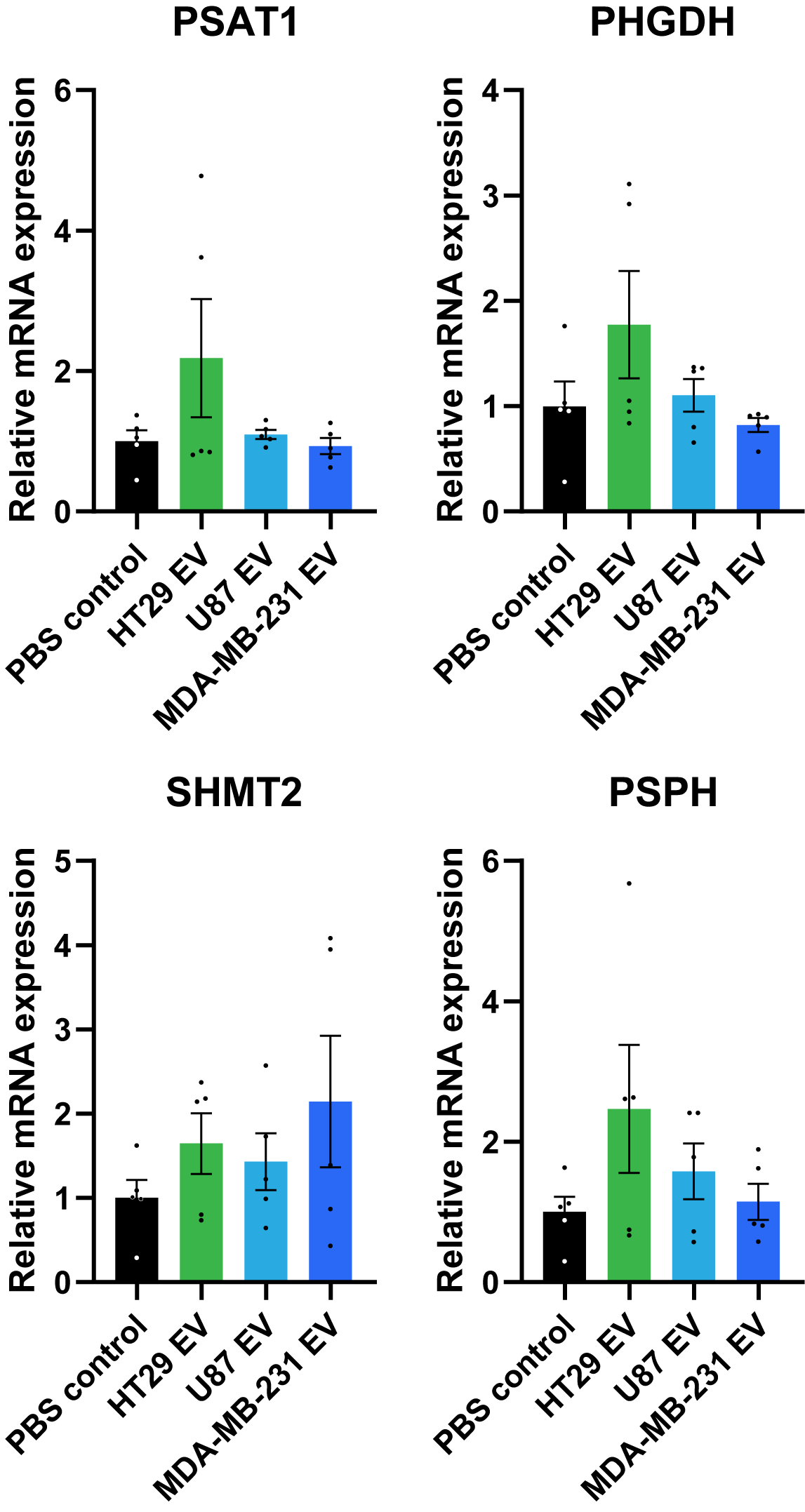


***Supplementary Figure 8: Cancer EV do not significantly alter serine/glycine synthesis pathway enzymes.*** *Expression of PSAT1, PHGDH, SHMT2 and PSPH in endothelial cells (HUVEC) were examined after 24 hour stimulation with HT29, U87 or MDA-MD-231 derived EV. N=5, mean ± SEM.*


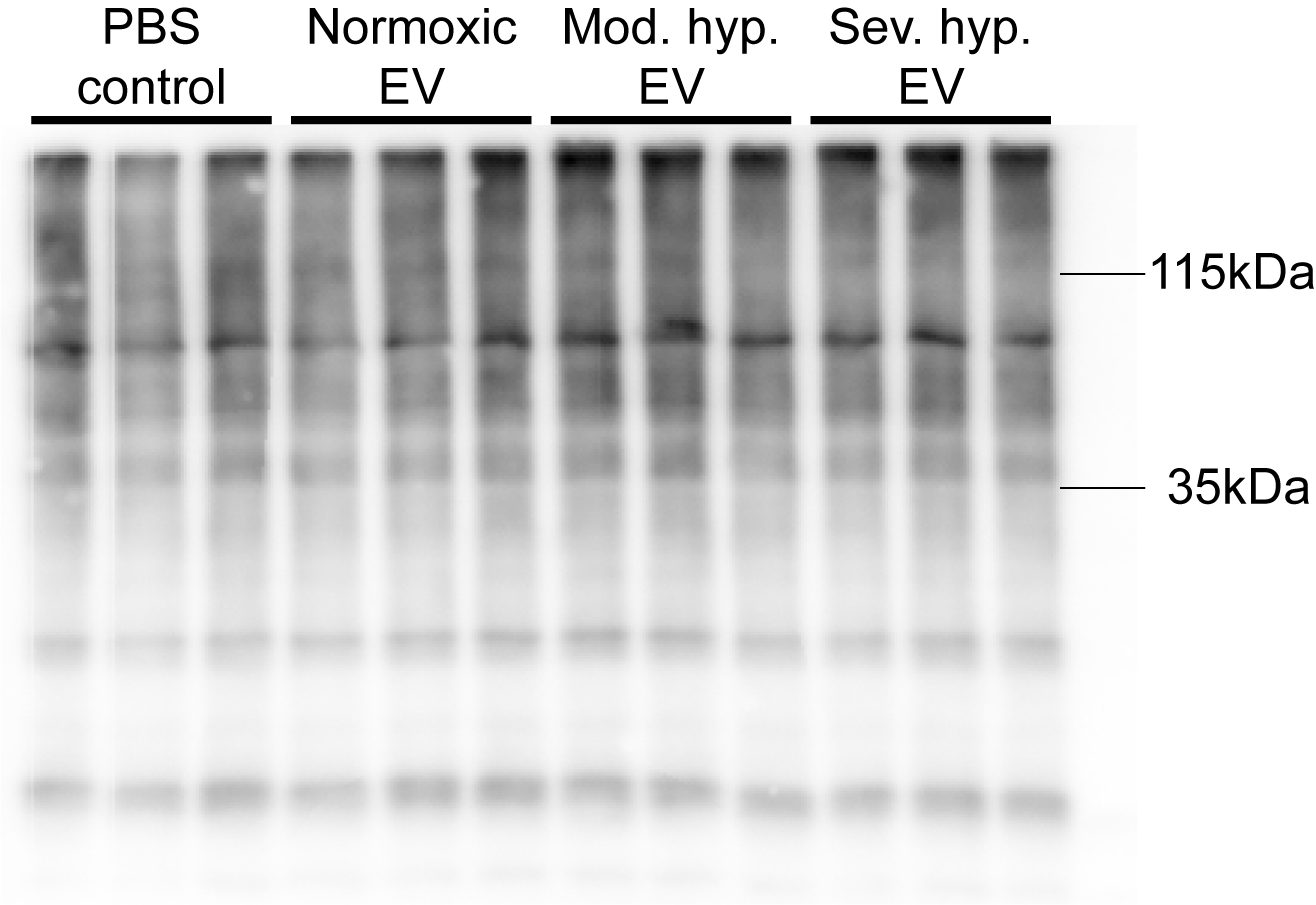


***Supplementary Figure 9: HT29 derived EV stimulate nascent protein synthesis***. *Immunoblot analysis of newly synthesized proteins in endothelial cells (HUVEC) after HT29 EV stimulation. Newly synthesized proteins were labelled with biotin using click-it chemistry and visualized using streptavidin-HRP, n=3.*


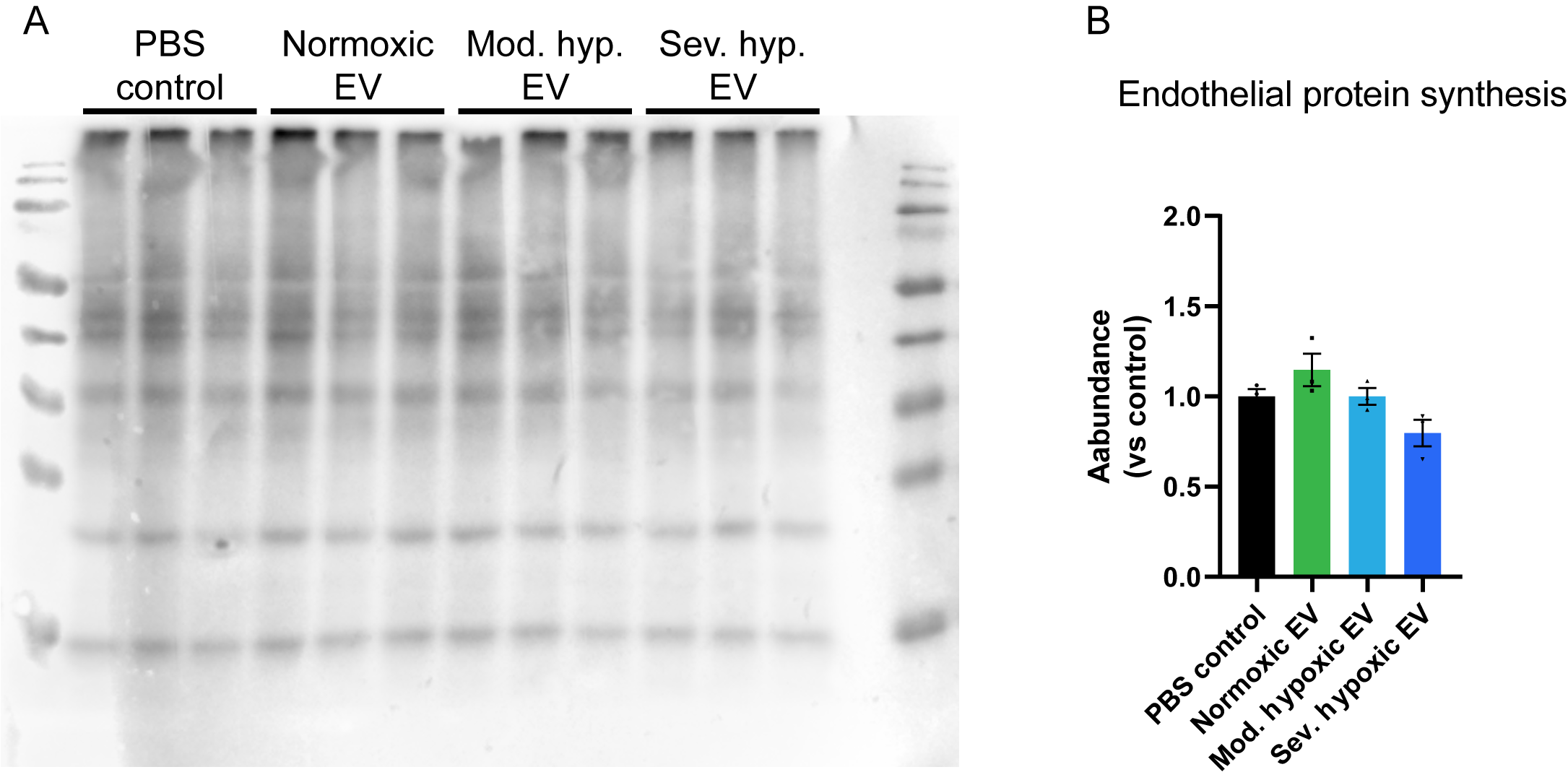


***Supplementary Figure 10: U87 derived EV do not stimulate nascent protein synthesis.*** *De Novo protein synthesis by endothelial cells (HUVEC) after stimulation with U87 derived EV was analyzed using click-it chemistry. Newly synthesized proteins were labelled with biotin and visualized using streptavidin-HRP. A)* *Immunoblot analysis of newly synthesized proteins in endothelial cells after U87 EV stimulation. B) Endothelial cell de novo protein synthesis upon U87 derived EV stimulation is not significantly altered, n=3, mean ± SEM.*


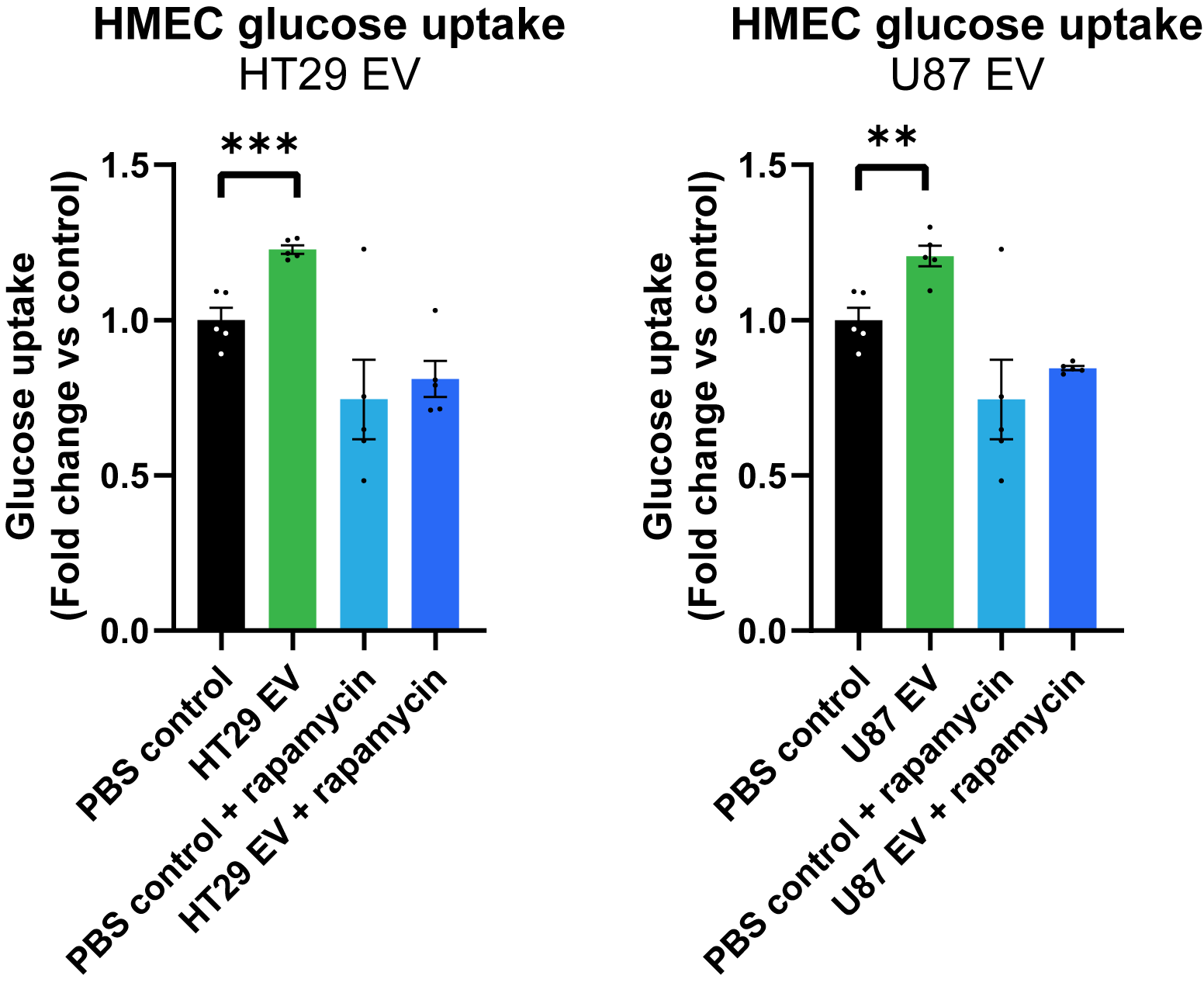


***Supplementary Figure 11: Rapamycin inhibits cancer EV induced glucose consumption in HMECs.*** *HT29 and U87 derived EV significantly increase glucose consumption in human microvascular endothelial cells (HMECs). Addition of rapamycin abolishes EV-induced glucose consumption. Unpaired t test, n=5, mean ± SEM, ** p<0.01 vs control, ***p<0.001 vs control.*
