# Supplementary material for: Cancer EV stimulate endothelial glycolysis to fuel protein synthesis via mTOR and AMPKα activation
Source: J Extracell Vesicles. 2024 Jul 13;13(7):e12449. doi: 10.1002/jev2.12449 (PMC11245686; doi:10.1002/jev2.12449)
Supplement: Supplementary file 2 — Supporting Information [file JEV2-13-e12449-s002.docx]

| **Target** | **Forward primer** | **Reverse primer** |
| --- | --- | --- |
| PSAT1 | AAAAACAATGGAGGTGCCGC | GGCTCCACTGGACAAACGTA |
| PHGDH | CCTCAATGTCACCACCTCCC | CATTGAGCCCCTGCAGTACA |
| SHMT2 | CTCTTTGTTTTGGGCGGCTC | GACACTGCCTGTCCTTCTCC |
| PSPH | TGTCAGAAATGACACGGCGA | GGGGGTTGCTCTGCTATGAG |
| RPL13A | CGA GGT TGG CTG GAA GTA CC | CTT CTC GGC CTG TTT CCG TAG |

***Supplementary table 1: Primer sequences for RT-qPCR.***

| **Accession** | **Description** | **Score Sequest HT** | **# PSMs** |
| --- | --- | --- | --- |
|  |  |  |  |
| O00468-6 | Isoform 6 of Agrin [OS=Homo sapiens] | 3616,93 | 1284 |
| P98160 | Basement membrane-specific heparan sulfate proteoglycan core protein [OS=Homo sapiens] | 2668,02 | 1021 |
| P19823 | Inter-alpha-trypsin inhibitor heavy chain H2 [OS=Homo sapiens] | 1415,43 | 535 |
| P60709 | Actin, cytoplasmic 1 [OS=Homo sapiens] | 1041,26 | 363 |
| P11142-1 | Heat shock cognate 71 kDa protein [OS=Homo sapiens] | 849,53 | 318 |
| Q08380 | Galectin-3-binding protein [OS=Homo sapiens] | 791,83 | 281 |
| P01023 | alpha-2-macroglobulin [OS=Homo sapiens] | 786,37 | 429 |
| P02765 | Alpha-2-HS-glycoprotein [OS=Homo sapiens] | 645,58 | 261 |
| P62736 | Actin, aortic smooth muscle [OS=Homo sapiens] | 599,86 | 239 |
| P04264 | Keratin, type II cytoskeletal 1 [OS=Homo sapiens] | 507,36 | 194 |
| O15230 | Laminin subunit alpha-5 [OS=Homo sapiens] | 462,17 | 223 |
| Q06033-1 | Inter-alpha-trypsin inhibitor heavy chain H3 [OS=Homo sapiens] | 430,8 | 238 |
| Q8WUM4-2 | Isoform 2 of Programmed cell death 6-interacting protein [OS=Homo sapiens] | 402,71 | 191 |
| O00560-1 | Syntenin-1 [OS=Homo sapiens] | 354,48 | 137 |
| P07996 | thrombospondin-1 [OS=Homo sapiens] | 344,7 | 148 |
| Q9GZM7-1 | Tubulointerstitial nephritis antigen-like [OS=Homo sapiens] | 314,27 | 105 |
| P20742 | Pregnancy zone protein [OS=Homo sapiens] | 295,52 | 152 |
| P0DMV8 | heat shock 70 kDa protein 1A [OS=Homo sapiens] | 283,22 | 105 |
| P11047 | Laminin subunit gamma-1 [OS=Homo sapiens] | 269,75 | 137 |
| P23142 | Fibulin-1 [OS=Homo sapiens] | 246,39 | 149 |
| P04004 | Vitronectin [OS=Homo sapiens] | 240,29 | 81 |
| P04114 | apolipoprotein B-100 [OS=Homo sapiens] | 222,65 | 127 |
| P23142-4 | Isoform C of Fibulin-1 [OS=Homo sapiens] | 200,88 | 130 |
| P35527 | Keratin, type I cytoskeletal 9 [OS=Homo sapiens] | 199,22 | 81 |
| P13645 | Keratin, type I cytoskeletal 10 [OS=Homo sapiens] | 196,49 | 94 |
| Q2PPJ7-1 | Ral GTPase-activating protein subunit alpha-2 [OS=Homo sapiens] | 194,64 | 132 |
| P16402 | Histone H1.3 [OS=Homo sapiens] | 186,28 | 78 |
| P07942 | Laminin subunit beta-1 [OS=Homo sapiens] | 185,25 | 94 |
| P16112 | Aggrecan core protein [OS=Homo sapiens] | 184,2 | 70 |
| P05556-1 | Integrin beta-1 [OS=Homo sapiens] | 175,8 | 88 |
| P13611 | Versican core protein [OS=Homo sapiens] | 173,71 | 63 |
| P21926 | CD9 antigen [OS=Homo sapiens] | 159,76 | 41 |
| P02771 | Alpha-fetoprotein [OS=Homo sapiens] | 141,7 | 60 |
| Q96JB6 | Lysyl oxidase homolog 4 [OS=Homo sapiens] | 138,58 | 52 |
| P23229-1 | integrin alpha-6 [OS=Homo sapiens] | 130,3 | 57 |
| P05023 | Sodium/potassium-transporting ATPase subunit alpha-1 [OS=Homo sapiens] | 126,42 | 66 |
| P16144-1 | Integrin beta-4 [OS=Homo sapiens] | 122,46 | 67 |
| P19827-1 | Inter-alpha-trypsin inhibitor heavy chain H1 [OS=Homo sapiens] | 118,01 | 46 |
| P02452 | Collagen alpha-1(I) chain [OS=Homo sapiens] | 96,73 | 47 |
| P31431-1 | syndecan-4 [OS=Homo sapiens] | 95,4 | 45 |
| P01024 | Complement C3 [OS=Homo sapiens] | 94,23 | 51 |
| P68104 | Elongation factor 1-alpha 1 [OS=Homo sapiens] | 94,21 | 38 |
| P02751-15 | Isoform 15 of Fibronectin [OS=Homo sapiens] | 93,19 | 58 |
| P08697-1 | Alpha-2-antiplasmin [OS=Homo sapiens] | 91,85 | 36 |
| P21333 | Filamin-A [OS=Homo sapiens] | 89,15 | 51 |
| Q6PCB0 | von Willebrand factor A domain-containing protein 1 [OS=Homo sapiens] | 88,29 | 34 |
| P01042 | kininogen-1 [OS=Homo sapiens] | 82,96 | 32 |
| P02533 | Keratin, type I cytoskeletal 14 [OS=Homo sapiens] | 80,31 | 46 |
| O43707 | Alpha-actinin-4 [OS=Homo sapiens] | 77,56 | 34 |
| P14923 | Junction plakoglobin [OS=Homo sapiens] | 74,69 | 30 |
| P68363 | Tubulin alpha-1B chain [OS=Homo sapiens] | 70,98 | 22 |
| P20929-2 | Isoform 2 of Nebulin [OS=Homo sapiens] | 64,84 | 37 |
| P16422 | Epithelial cell adhesion molecule [OS=Homo sapiens] | 63,31 | 30 |
| P16401 | Histone H1.5 [OS=Homo sapiens] | 59,57 | 34 |
| P07355-2 | Isoform 2 of Annexin A2 [OS=Homo sapiens] | 57,49 | 25 |
| P0C0L4-1 | Complement C4-A [OS=Homo sapiens] | 53,04 | 29 |
| P02786 | Transferrin receptor protein 1 [OS=Homo sapiens] | 49,32 | 17 |
| P00734 | Prothrombin [OS=Homo sapiens] | 48,69 | 29 |
| P08754 | Guanine nucleotide-binding protein G(k) subunit alpha [OS=Homo sapiens] | 47 | 24 |
| P51884 | Lumican [OS=Homo sapiens] | 46,84 | 32 |
| Q14517 | Protocadherin Fat 1 [OS=Homo sapiens] | 45,87 | 22 |
| Q15758-1 | Neutral amino acid transporter B(0) [OS=Homo sapiens] | 41,65 | 18 |
| P61224-1 | Ras-related protein Rap-1b [OS=Homo sapiens] | 40,27 | 15 |
| P35221-2 | Isoform 2 of Catenin alpha-1 [OS=Homo sapiens] | 39,82 | 27 |
| P04075-2 | Isoform 2 of Fructose-bisphosphate aldolase A [OS=Homo sapiens] | 39,58 | 20 |
| P14618 | Pyruvate kinase PKM [OS=Homo sapiens] | 38,54 | 11 |
| Q9Y490 | Talin-1 [OS=Homo sapiens] | 37,19 | 21 |
| Q5JWF2-1 | Guanine nucleotide-binding protein G(S) subunit alpha isoforms XLas [OS=Homo sapiens] | 36,7 | 19 |
| O95497 | Pantetheinase [OS=Homo sapiens] | 35,11 | 24 |
| P16070 | CD44 antigen [OS=Homo sapiens] | 34,79 | 17 |
| P62873 | Guanine nucleotide-binding protein G(I)/G(S)/G(T) subunit beta-1 [OS=Homo sapiens] | 34,01 | 23 |
| P35579-1 | Myosin-9 [OS=Homo sapiens] | 33,24 | 16 |
| P35443 | Thrombospondin-4 [OS=Homo sapiens] | 33,13 | 26 |
| P07477 | Trypsin-1 [OS=Homo sapiens] | 32,22 | 13 |
| P62879 | Guanine nucleotide-binding protein G(I)/G(S)/G(T) subunit beta-2 [OS=Homo sapiens] | 31,81 | 22 |
| Q9P2B2 | prostaglandin F2 receptor negative regulator [OS=Homo sapiens] | 30,42 | 17 |
| P68431 | Histone H3.1 [OS=Homo sapiens] | 28,37 | 15 |
| P35613 | Basigin [OS=Homo sapiens] | 27,55 | 15 |
| P05106 | Integrin beta-3 [OS=Homo sapiens] | 26,97 | 11 |
| O15551 | Claudin-3 [OS=Homo sapiens] | 26,94 | 23 |
| P46940 | Ras GTPase-activating-like protein IQGAP1 [OS=Homo sapiens] | 26,76 | 16 |
| Q86X29 | Lipolysis-stimulated lipoprotein receptor [OS=Homo sapiens] | 26,58 | 15 |
| P26038 | Moesin [OS=Homo sapiens] | 26,37 | 17 |
| P62937 | peptidyl-prolyl cis-trans isomerase A [OS=Homo sapiens] | 26,2 | 17 |
| P60953 | Cell division control protein 42 homolog [OS=Homo sapiens] | 26,12 | 13 |
| Q16787-2 | Laminin subunit alpha-3 [OS=Homo sapiens] | 26,12 | 17 |
| P00747 | Plasminogen [OS=Homo sapiens] | 26,08 | 16 |
| P02538 | Keratin, type II cytoskeletal 6A [OS=Homo sapiens] | 24,9 | 17 |
| P36578 | 60S ribosomal protein L4 [OS=Homo sapiens] | 24,84 | 18 |
| P02649 | Apolipoprotein E [OS=Homo sapiens] | 24,73 | 11 |
| P13647 | keratin, type II cytoskeletal 5 [OS=Homo sapiens] | 24,47 | 18 |
| Q96CF2 | Charged multivesicular body protein 4c [OS=Homo sapiens] | 23,84 | 34 |
| P08195-4 | Isoform 4 of 4F2 cell-surface antigen heavy chain [OS=Homo sapiens] | 23,79 | 9 |
| Q14697-2 | Isoform 2 of Neutral alpha-glucosidase AB [OS=Homo sapiens] | 22,97 | 8 |
| P36955 | Pigment epithelium-derived factor [OS=Homo sapiens] | 22,06 | 9 |
| P63000-2 | Isoform B of Ras-related C3 botulinum toxin substrate 1 [OS=Homo sapiens] | 21,97 | 14 |
| P08238 | Heat shock protein HSP 90-beta [OS=Homo sapiens] | 21,97 | 10 |
| P60033 | CD81 antigen [OS=Homo sapiens] | 21,04 | 5 |
| P09758 | Tumor-associated calcium signal transducer 2 [OS=Homo sapiens] | 20,72 | 7 |
| O95395 | Beta-1,3-galactosyl-O-glycosyl-glycoprotein beta-1,6-N-acetylglucosaminyltransferase 3 [OS=Homo sapiens] | 20,57 | 9 |
| P26006-1 | Isoform 2 of Integrin alpha-3 [OS=Homo sapiens] | 19,64 | 17 |
| P07195 | L-lactate dehydrogenase B chain [OS=Homo sapiens] | 19,57 | 15 |
| P00533-1 | epidermal growth factor receptor [OS=Homo sapiens] | 19,52 | 13 |
| O14672 | Disintegrin and metalloproteinase domain-containing protein 10 [OS=Homo sapiens] | 18,63 | 17 |
| P13987 | CD59 glycoprotein [OS=Homo sapiens] | 18,54 | 14 |
| P07900-2 | Isoform 2 of Heat shock protein HSP 90-alpha [OS=Homo sapiens] | 18,49 | 11 |
| O75369-8 | Isoform 8 of Filamin-B [OS=Homo sapiens] | 18,48 | 13 |
| O60716-1 | Catenin delta-1 [OS=Homo sapiens] | 17,73 | 10 |
| Q5QNW6-2 | Isoform 2 of Histone H2B type 2-F [OS=Homo sapiens] | 16,51 | 15 |
| P11166 | Solute carrier family 2, facilitated glucose transporter member 1 [OS=Homo sapiens] | 16,41 | 10 |
| P02461 | Collagen alpha-1(III) chain [OS=Homo sapiens] | 16,32 | 18 |
| O00159-1 | Unconventional myosin-Ic [OS=Homo sapiens] | 15,88 | 6 |
| P55290-4 | Isoform 4 of Cadherin-13 [OS=Homo sapiens] | 15,73 | 7 |
| P10643 | Complement component C7 [OS=Homo sapiens] | 15,64 | 9 |
| P12109 | Collagen alpha-1(VI) chain [OS=Homo sapiens] | 14,91 | 14 |
| Q16513 | Serine/threonine-protein kinase N2 [OS=Homo sapiens] | 14,74 | 8 |
| Q86VE9-4 | Isoform 4 of Serine incorporator 5 [OS=Homo sapiens] | 14,65 | 6 |
| P98088 | Mucin-5AC [OS=Homo sapiens] | 13,89 | 6 |
| P23528 | Cofilin-1 [OS=Homo sapiens] | 13,75 | 13 |
| P60660 | Myosin light polypeptide 6 [OS=Homo sapiens] | 13,39 | 7 |
| P03951 | Coagulation factor XI [OS=Homo sapiens] | 13,19 | 8 |
| P05026 | Sodium/potassium-transporting ATPase subunit beta-1 [OS=Homo sapiens] | 12,91 | 6 |
| Q15262-4 | Isoform 4 of Receptor-type tyrosine-protein phosphatase kappa [OS=Homo sapiens] | 11,69 | 6 |
| P01130 | Low-density lipoprotein receptor [OS=Homo sapiens] | 11,35 | 6 |
| Q6UVK1 | Chondroitin sulfate proteoglycan 4 [OS=Homo sapiens] | 11,19 | 7 |
| O60635 | Tetraspanin-1 [OS=Homo sapiens] | 10,88 | 4 |
| P19075 | Tetraspanin-8 [OS=Homo sapiens] | 10,85 | 6 |
| P48509 | CD151 antigen [OS=Homo sapiens] | 10,79 | 10 |
| Q9H4B7 | tubulin beta-1 chain [OS=Homo sapiens] | 10,72 | 5 |
| P60174 | Triosephosphate isomerase [OS=Homo sapiens] | 10,65 | 6 |
| P29317 | Ephrin type-A receptor 2 [OS=Homo sapiens] | 10,58 | 13 |
| P07737 | profilin-1 [OS=Homo sapiens] | 10,48 | 4 |
| P35052 | Glypican-1 [OS=Homo sapiens] | 10,13 | 6 |
| Q8NG11-1 | Tetraspanin-14 [OS=Homo sapiens] | 10,08 | 5 |
| P35222 | Catenin beta-1 [OS=Homo sapiens] | 9,92 | 5 |
| Q969P0 | Immunoglobulin superfamily member 8 [OS=Homo sapiens] | 9,54 | 6 |
| B9A064-1 | Immunoglobulin lambda-like polypeptide 5 [OS=Homo sapiens] | 9,51 | 5 |
| Q06828 | Fibromodulin [OS=Homo sapiens] | 9,28 | 6 |
| O75144-2 | Isoform 2 of ICOS ligand [OS=Homo sapiens] | 9,12 | 5 |
| P17301 | Integrin alpha-2 [OS=Homo sapiens] | 9,06 | 4 |
| Q06710-3 | Isoform 3 of Paired box protein Pax-8 [OS=Homo sapiens] | 8,85 | 10 |
| O60884 | DnaJ homolog subfamily A member 2 [OS=Homo sapiens] | 8,79 | 5 |
| Q9Y5Y6 | suppressor of tumorigenicity 14 protein [OS=Homo sapiens] | 8,62 | 5 |
| Q14624-1 | Inter-alpha-trypsin inhibitor heavy chain H4 [OS=Homo sapiens] | 8,3 | 3 |
| Q9P265 | Disco-interacting protein 2 homolog B [OS=Homo sapiens] | 8,19 | 5 |
| P63104-1 | 14-3-3 protein zeta/delta [OS=Homo sapiens] | 7,8 | 6 |
| P08123 | Collagen alpha-2(I) chain [OS=Homo sapiens] | 7,15 | 7 |
| P39023 | 60S ribosomal protein L3 [OS=Homo sapiens] | 7,08 | 9 |
| P08514-1 | integrin alpha-IIb [OS=Homo sapiens] | 6,93 | 3 |
| P61981 | 14-3-3 protein gamma [OS=Homo sapiens] | 6,73 | 8 |
| Q06830 | peroxiredoxin-1 [OS=Homo sapiens] | 6,63 | 3 |
| Q9H4G4 | Golgi-associated plant pathogenesis-related protein 1 [OS=Homo sapiens] | 6,61 | 3 |
| P68371 | Tubulin beta-4B chain [OS=Homo sapiens] | 6,5 | 4 |
| Q9Y624 | Junctional adhesion molecule A [OS=Homo sapiens] | 6,23 | 6 |
| Q07020 | 60S ribosomal protein L18 [OS=Homo sapiens] | 6,14 | 3 |
| P12259 | Coagulation factor V [OS=Homo sapiens] | 6,04 | 2 |
| Q8WV92 | MIT domain-containing protein 1 [OS=Homo sapiens] | 5,95 | 2 |
| O94985-1 | Calsyntenin-1 [OS=Homo sapiens] | 5,94 | 7 |
| Q9UKE5-1 | Traf2 and NCK-interacting protein kinase [OS=Homo sapiens] | 5,87 | 3 |
| P06744-2 | Isoform 2 of Glucose-6-phosphate isomerase [OS=Homo sapiens] | 5,79 | 3 |
| P98172 | ephrin-B1 [OS=Homo sapiens] | 5,67 | 2 |
| P00558 | phosphoglycerate kinase 1 [OS=Homo sapiens] | 5,45 | 4 |
| P02774-3 | Isoform 3 of Vitamin D-binding protein [OS=Homo sapiens] | 5,35 | 5 |
| P15880 | 40S ribosomal protein S2 [OS=Homo sapiens] | 5,17 | 3 |

***Supplementary table 2: Mass spectrometry analysis of proteins in HT29 derived EV.***

| **Accession** | **Description** | **Score Sequest HT** | **# PSMs** |
| --- | --- | --- | --- |
|  |  |  |  |
| P13611-2 | Isoform V1 of Versican core protein [OS=Homo sapiens] | 4057,71 | 1452 |
| P13611-5 | Isoform Vint of Versican core protein [OS=Homo sapiens] | 3872,87 | 1362 |
| P19823 | Inter-alpha-trypsin inhibitor heavy chain H2 [OS=Homo sapiens] | 2073,51 | 797 |
| P05556-1 | Integrin beta-1 [OS=Homo sapiens] | 1540,06 | 559 |
| P24821 | Tenascin [OS=Homo sapiens] | 1142,6 | 424 |
| P60709 | Actin, cytoplasmic 1 [OS=Homo sapiens] | 1101,54 | 370 |
| P01023 | alpha-2-macroglobulin [OS=Homo sapiens] | 1009,91 | 561 |
| P12111 | Collagen alpha-3(VI) chain [OS=Homo sapiens] | 968,56 | 397 |
| P23142-4 | Isoform C of Fibulin-1 [OS=Homo sapiens] | 919 | 363 |
| P02765 | Alpha-2-HS-glycoprotein [OS=Homo sapiens] | 868,43 | 320 |
| P02751-15 | Isoform 15 of Fibronectin [OS=Homo sapiens] | 803,01 | 332 |
| P11142-1 | Heat shock cognate 71 kDa protein [OS=Homo sapiens] | 798,91 | 316 |
| P26006 | Integrin alpha-3 [OS=Homo sapiens] | 787,98 | 354 |
| P23142 | Fibulin-1 [OS=Homo sapiens] | 782,48 | 315 |
| Q06033-1 | Inter-alpha-trypsin inhibitor heavy chain H3 [OS=Homo sapiens] | 752,84 | 281 |
| P21589-1 | 5'-nucleotidase [OS=Homo sapiens] | 686,37 | 250 |
| P05023 | Sodium/potassium-transporting ATPase subunit alpha-1 [OS=Homo sapiens] | 664,22 | 270 |
| P04264 | Keratin, type II cytoskeletal 1 [OS=Homo sapiens] | 634,2 | 238 |
| Q08380 | Galectin-3-binding protein [OS=Homo sapiens] | 618,66 | 221 |
| P62736 | Actin, aortic smooth muscle [OS=Homo sapiens] | 581,07 | 238 |
| P02452 | Collagen alpha-1(I) chain [OS=Homo sapiens] | 515,27 | 249 |
| O00468-7 | Isoform 7 of Agrin [OS=Homo sapiens] | 467,4 | 202 |
| P16070 | CD44 antigen [OS=Homo sapiens] | 465,9 | 223 |
| P26022 | Pentraxin-related protein PTX3 [OS=Homo sapiens] | 454,21 | 156 |
| Q9P2B2 | prostaglandin F2 receptor negative regulator [OS=Homo sapiens] | 419,86 | 180 |
| P11047 | Laminin subunit gamma-1 [OS=Homo sapiens] | 392,17 | 176 |
| P20742 | Pregnancy zone protein [OS=Homo sapiens] | 389,2 | 178 |
| P07942 | Laminin subunit beta-1 [OS=Homo sapiens] | 378,76 | 172 |
| P35527 | Keratin, type I cytoskeletal 9 [OS=Homo sapiens] | 364,32 | 149 |
| P08123 | Collagen alpha-2(I) chain [OS=Homo sapiens] | 363,25 | 126 |
| O15230 | Laminin subunit alpha-5 [OS=Homo sapiens] | 342,83 | 160 |
| P04004 | Vitronectin [OS=Homo sapiens] | 308,84 | 122 |
| P68104 | Elongation factor 1-alpha 1 [OS=Homo sapiens] | 307,6 | 122 |
| P01042 | kininogen-1 [OS=Homo sapiens] | 306,29 | 111 |
| P04899-4 | Isoform sGi2 of Guanine nucleotide-binding protein G(i) subunit alpha-2 [OS=Homo sapiens] | 304,97 | 114 |
| P12109 | Collagen alpha-1(VI) chain [OS=Homo sapiens] | 294,4 | 126 |
| P62879 | Guanine nucleotide-binding protein G(I)/G(S)/G(T) subunit beta-2 [OS=Homo sapiens] | 287,96 | 117 |
| P16112 | Aggrecan core protein [OS=Homo sapiens] | 252,17 | 91 |
| P04114 | apolipoprotein B-100 [OS=Homo sapiens] | 233,63 | 132 |
| P02771 | Alpha-fetoprotein [OS=Homo sapiens] | 221,8 | 83 |
| P62873 | Guanine nucleotide-binding protein G(I)/G(S)/G(T) subunit beta-1 [OS=Homo sapiens] | 209,82 | 94 |
| P04406-1 | glyceraldehyde-3-phosphate dehydrogenase [OS=Homo sapiens] | 193,04 | 65 |
| Q9HAV0 | Guanine nucleotide-binding protein subunit beta-4 [OS=Homo sapiens] | 171,12 | 79 |
| P04075-2 | Isoform 2 of Fructose-bisphosphate aldolase A [OS=Homo sapiens] | 162,77 | 58 |
| P14618 | Pyruvate kinase PKM [OS=Homo sapiens] | 162,16 | 51 |
| P07996 | thrombospondin-1 [OS=Homo sapiens] | 151,74 | 65 |
| P13987 | CD59 glycoprotein [OS=Homo sapiens] | 143,68 | 68 |
| P08754 | Guanine nucleotide-binding protein G(k) subunit alpha [OS=Homo sapiens] | 140,87 | 71 |
| P08648 | Integrin alpha-5 [OS=Homo sapiens] | 138,43 | 61 |
| P01024 | Complement C3 [OS=Homo sapiens] | 132,24 | 82 |
| P19827-1 | Inter-alpha-trypsin inhibitor heavy chain H1 [OS=Homo sapiens] | 129,95 | 72 |
| P62937 | peptidyl-prolyl cis-trans isomerase A [OS=Homo sapiens] | 128,66 | 72 |
| P35613 | Basigin [OS=Homo sapiens] | 117,4 | 41 |
| P17301 | Integrin alpha-2 [OS=Homo sapiens] | 115,73 | 54 |
| P07900-2 | Isoform 2 of Heat shock protein HSP 90-alpha [OS=Homo sapiens] | 112,23 | 51 |
| O00560-1 | Syntenin-1 [OS=Homo sapiens] | 111,47 | 51 |
| P02533 | Keratin, type I cytoskeletal 14 [OS=Homo sapiens] | 106,78 | 51 |
| P08238 | Heat shock protein HSP 90-beta [OS=Homo sapiens] | 104,77 | 41 |
| P09382 | Galectin-1 [OS=Homo sapiens] | 101,39 | 31 |
| Q96Q89-2 | Isoform 2 of Kinesin-like protein KIF20B [OS=Homo sapiens] | 98,3 | 60 |
| P08779 | Keratin, type I cytoskeletal 16 [OS=Homo sapiens] | 96,96 | 50 |
| P68363 | Tubulin alpha-1B chain [OS=Homo sapiens] | 96,93 | 35 |
| P12110 | Collagen alpha-2(VI) chain [OS=Homo sapiens] | 95,51 | 47 |
| P01892 | HLA class I histocompatibility antigen, A-2 alpha chain [OS=Homo sapiens] | 93,51 | 52 |
| Q15582 | Transforming growth factor-beta-induced protein ig-h3 [OS=Homo sapiens] | 91,72 | 41 |
| P02795 | metallothionein-2 [OS=Homo sapiens] | 89,98 | 43 |
| P08697-1 | Alpha-2-antiplasmin [OS=Homo sapiens] | 88,96 | 34 |
| P10909-2 | Isoform 2 of Clusterin [OS=Homo sapiens] | 86,63 | 52 |
| P06733-1 | alpha-enolase [OS=Homo sapiens] | 85,64 | 56 |
| Q5JWF2-1 | Guanine nucleotide-binding protein G(S) subunit alpha isoforms XLas [OS=Homo sapiens] | 85,5 | 39 |
| P48509 | CD151 antigen [OS=Homo sapiens] | 84,44 | 40 |
| O14786-1 | Neuropilin-1 [OS=Homo sapiens] | 83,11 | 41 |
| P0DMV8 | heat shock 70 kDa protein 1A [OS=Homo sapiens] | 82,88 | 35 |
| P60174 | Triosephosphate isomerase [OS=Homo sapiens] | 82,26 | 40 |
| P04259 | keratin, type II cytoskeletal 6B [OS=Homo sapiens] | 80,4 | 39 |
| P00533-1 | epidermal growth factor receptor [OS=Homo sapiens] | 73,2 | 47 |
| P51884 | Lumican [OS=Homo sapiens] | 73,15 | 49 |
| P0C0L4-1 | Complement C4-A [OS=Homo sapiens] | 70,79 | 34 |
| P02461 | Collagen alpha-1(III) chain [OS=Homo sapiens] | 69,93 | 33 |
| P02538 | Keratin, type II cytoskeletal 6A [OS=Homo sapiens] | 69,43 | 35 |
| Q14766-4 | Isoform 4 of Latent-transforming growth factor beta-binding protein 1 [OS=Homo sapiens] | 66,47 | 42 |
| P22105-4 | Isoform 5 of Tenascin-X [OS=Homo sapiens] | 65,23 | 32 |
| P00747 | Plasminogen [OS=Homo sapiens] | 63,25 | 35 |
| P15153 | Ras-related C3 botulinum toxin substrate 2 [OS=Homo sapiens] | 63,09 | 44 |
| P35555 | Fibrillin-1 [OS=Homo sapiens] | 62,39 | 45 |
| P09603 | Macrophage colony-stimulating factor 1 [OS=Homo sapiens] | 58,62 | 17 |
| A1L4H1-1 | Soluble scavenger receptor cysteine-rich domain-containing protein SSC5D [OS=Homo sapiens] | 58,08 | 34 |
| P07737 | profilin-1 [OS=Homo sapiens] | 57,39 | 22 |
| P27105 | erythrocyte band 7 integral membrane protein [OS=Homo sapiens] | 56,96 | 30 |
| P00734 | Prothrombin [OS=Homo sapiens] | 53,24 | 23 |
| P00338-3 | Isoform 3 of L-lactate dehydrogenase A chain [OS=Homo sapiens] | 49,99 | 27 |
| P13647 | keratin, type II cytoskeletal 5 [OS=Homo sapiens] | 49,92 | 29 |
| P07195 | L-lactate dehydrogenase B chain [OS=Homo sapiens] | 49,76 | 35 |
| P35443 | Thrombospondin-4 [OS=Homo sapiens] | 49,55 | 38 |
| P63000-2 | Isoform B of Ras-related C3 botulinum toxin substrate 1 [OS=Homo sapiens] | 46,72 | 31 |
| Q06828 | Fibromodulin [OS=Homo sapiens] | 46,15 | 23 |
| Q99536 | Synaptic vesicle membrane protein VAT-1 homolog [OS=Homo sapiens] | 45,53 | 14 |
| P13639 | Elongation factor 2 [OS=Homo sapiens] | 45,44 | 23 |
| Q9Y490 | Talin-1 [OS=Homo sapiens] | 43,9 | 28 |
| P00558 | phosphoglycerate kinase 1 [OS=Homo sapiens] | 43,27 | 22 |
| P60953 | Cell division control protein 42 homolog [OS=Homo sapiens] | 42,69 | 20 |
| P55290-4 | Isoform 4 of Cadherin-13 [OS=Homo sapiens] | 42,6 | 16 |
| P60033 | CD81 antigen [OS=Homo sapiens] | 42,42 | 12 |
| P06756 | Integrin alpha-V [OS=Homo sapiens] | 40,26 | 24 |
| O95497 | Pantetheinase [OS=Homo sapiens] | 39,67 | 24 |
| P20020 | Plasma membrane calcium-transporting ATPase 1 [OS=Homo sapiens] | 38,23 | 18 |
| Q2PPJ7-1 | Ral GTPase-activating protein subunit alpha-2 [OS=Homo sapiens] | 37,06 | 53 |
| P23528 | Cofilin-1 [OS=Homo sapiens] | 33,95 | 36 |
| P36955 | Pigment epithelium-derived factor [OS=Homo sapiens] | 33,44 | 13 |
| O43854-1 | EGF-like repeat and discoidin I-like domain-containing protein 3 [OS=Homo sapiens] | 28,85 | 13 |
| P63104-1 | 14-3-3 protein zeta/delta [OS=Homo sapiens] | 27,73 | 19 |
| P21333 | Filamin-A [OS=Homo sapiens] | 27,62 | 20 |
| P05106 | Integrin beta-3 [OS=Homo sapiens] | 27,61 | 14 |
| P05543 | thyroxine-binding globulin [OS=Homo sapiens] | 26,51 | 12 |
| P84095 | Rho-related GTP-binding protein RhoG [OS=Homo sapiens] | 26,26 | 12 |
| Q8WUM4-2 | Isoform 2 of Programmed cell death 6-interacting protein [OS=Homo sapiens] | 25,95 | 20 |
| P61764-2 | Isoform 2 of Syntaxin-binding protein 1 [OS=Homo sapiens] | 25,45 | 12 |
| P29323 | Ephrin type-B receptor 2 [OS=Homo sapiens] | 24,86 | 15 |
| P02649 | Apolipoprotein E [OS=Homo sapiens] | 23,58 | 10 |
| P05156 | Complement factor I [OS=Homo sapiens] | 23,36 | 22 |
| O60488-1 | Long-chain-fatty-acid--CoA ligase 4 [OS=Homo sapiens] | 23,1 | 9 |
| Q16537 | Serine/threonine-protein phosphatase 2A 56 kDa regulatory subunit epsilon isoform [OS=Homo sapiens] | 22,81 | 12 |
| P61224-1 | Ras-related protein Rap-1b [OS=Homo sapiens] | 22,58 | 12 |
| Q96CF2 | Charged multivesicular body protein 4c [OS=Homo sapiens] | 22,43 | 21 |
| P10643 | Complement component C7 [OS=Homo sapiens] | 22,23 | 10 |
| P02749 | Beta-2-glycoprotein 1 [OS=Homo sapiens] | 21,88 | 11 |
| P20908 | Collagen alpha-1(V) chain [OS=Homo sapiens] | 21,51 | 11 |
| P26038 | Moesin [OS=Homo sapiens] | 21,44 | 10 |
| P02774-3 | Isoform 3 of Vitamin D-binding protein [OS=Homo sapiens] | 21,3 | 16 |
| P02753 | Retinol-binding protein 4 [OS=Homo sapiens] | 20,14 | 17 |
| O75144-2 | Isoform 2 of ICOS ligand [OS=Homo sapiens] | 20,04 | 11 |
| P05121-1 | Plasminogen activator inhibitor 1 [OS=Homo sapiens] | 19,68 | 7 |
| P62834 | ras-related protein Rap-1A [OS=Homo sapiens] | 19,16 | 11 |
| P21926 | CD9 antigen [OS=Homo sapiens] | 19,04 | 6 |
| Q969P0 | Immunoglobulin superfamily member 8 [OS=Homo sapiens] | 18,84 | 13 |
| Q9UBI6 | guanine nucleotide-binding protein g(i)/g(s)/g(o) subunit gamma-12 [OS=Homo sapiens] | 18,81 | 8 |
| P13693 | Translationally-controlled tumor protein [OS=Homo sapiens] | 18,48 | 16 |
| P52823 | Stanniocalcin-1 [OS=Homo sapiens] | 17,72 | 7 |
| Q06830 | peroxiredoxin-1 [OS=Homo sapiens] | 16,47 | 8 |
| Q92597 | Protein NDRG1 [OS=Homo sapiens] | 16,14 | 7 |
| P29317 | Ephrin type-A receptor 2 [OS=Homo sapiens] | 15,97 | 15 |
| Q9BY76 | Angiopoietin-related protein 4 [OS=Homo sapiens] | 15,27 | 7 |
| Q13740-1 | CD166 antigen [OS=Homo sapiens] | 15,21 | 15 |
| Q02388-1 | Collagen alpha-1(VII) chain [OS=Homo sapiens] | 15,16 | 13 |
| P08195-4 | Isoform 4 of 4F2 cell-surface antigen heavy chain [OS=Homo sapiens] | 15,09 | 6 |
| P61981 | 14-3-3 protein gamma [OS=Homo sapiens] | 14,77 | 13 |
| P27348 | 14-3-3 protein theta [OS=Homo sapiens] | 13,24 | 10 |
| Q07954 | prolow-density lipoprotein receptor-related protein 1 [OS=Homo sapiens] | 13,09 | 10 |
| O00159-1 | Unconventional myosin-Ic [OS=Homo sapiens] | 12,52 | 9 |
| Q16363 | Laminin subunit alpha-4 [OS=Homo sapiens] | 12,46 | 7 |
| P02766 | Transthyretin [OS=Homo sapiens] | 12,44 | 11 |
| Q14112-1 | Nidogen-2 [OS=Homo sapiens] | 12,05 | 5 |
| Q5QNW6-2 | Isoform 2 of Histone H2B type 2-F [OS=Homo sapiens] | 11,98 | 9 |
| P51153 | Ras-related protein Rab-13 [OS=Homo sapiens] | 11,95 | 10 |
| Q15262-4 | Isoform 4 of Receptor-type tyrosine-protein phosphatase kappa [OS=Homo sapiens] | 11,48 | 7 |
| B9A064-1 | Immunoglobulin lambda-like polypeptide 5 [OS=Homo sapiens] | 11,36 | 6 |
| P61026 | ras-related protein rab-10 [OS=Homo sapiens] | 11,21 | 9 |
| Q15043 | Zinc transporter ZIP14 [OS=Homo sapiens] | 10,83 | 5 |
| O00186 | syntaxin-binding protein 3 [OS=Homo sapiens] | 10,56 | 10 |
| Q02952-1 | A-kinase anchor protein 12 [OS=Homo sapiens] | 10,52 | 6 |
| Q7Z7G0 | Target of Nesh-SH3 [OS=Homo sapiens] | 10,43 | 8 |
| Q9C0B5 | Palmitoyltransferase ZDHHC5 [OS=Homo sapiens] | 9,85 | 6 |
| P07437 | tubulin beta chain [OS=Homo sapiens] | 9,57 | 4 |
| P11234-2 | Isoform 2 of Ras-related protein Ral-B [OS=Homo sapiens] | 9,33 | 5 |
| P19320-1 | Vascular cell adhesion protein 1 [OS=Homo sapiens] | 9,16 | 3 |
| P02786 | Transferrin receptor protein 1 [OS=Homo sapiens] | 9,06 | 3 |
| O00469-2 | Isoform 2 of Procollagen-lysine,2-oxoglutarate 5-dioxygenase 2 [OS=Homo sapiens] | 8,96 | 4 |
| P15121 | aldose reductase [OS=Homo sapiens] | 8,93 | 17 |
| Q04721 | Neurogenic locus notch homolog protein 2 [OS=Homo sapiens] | 8,92 | 5 |
| Q9H4M9 | EH domain-containing protein 1 [OS=Homo sapiens] | 8,89 | 3 |
| Q15758-1 | Neutral amino acid transporter B(0) [OS=Homo sapiens] | 8,7 | 5 |
| Q16270-1 | Insulin-like growth factor-binding protein 7 [OS=Homo sapiens] | 8,5 | 5 |
| P13591-2 | Neural cell adhesion molecule 1 [OS=Homo sapiens] | 8,45 | 5 |
| O14672 | Disintegrin and metalloproteinase domain-containing protein 10 [OS=Homo sapiens] | 8,27 | 7 |
| P18206 | Vinculin [OS=Homo sapiens] | 8,2 | 9 |
| Q6UVK1 | Chondroitin sulfate proteoglycan 4 [OS=Homo sapiens] | 7,81 | 5 |
| O00299 | chloride intracellular channel protein 1 [OS=Homo sapiens] | 7,72 | 6 |
| Q9H4G4 | Golgi-associated plant pathogenesis-related protein 1 [OS=Homo sapiens] | 7,67 | 3 |
| P07355-2 | Isoform 2 of Annexin A2 [OS=Homo sapiens] | 7,58 | 5 |
| Q9BXJ4-3 | Isoform 3 of Complement C1q tumor necrosis factor-related protein 3 [OS=Homo sapiens] | 7,38 | 3 |
| P30481 | HLA class I histocompatibility antigen, B-44 alpha chain [OS=Homo sapiens] | 7,13 | 7 |
| P47756-2 | Isoform 2 of F-actin-capping protein subunit beta [OS=Homo sapiens] | 7 | 5 |
| P21246 | Pleiotrophin [OS=Homo sapiens] | 6,16 | 3 |
| P40925-3 | Isoform 3 of Malate dehydrogenase, cytoplasmic [OS=Homo sapiens] | 6,11 | 3 |
| Q9Y6M5 | zinc transporter 1 [OS=Homo sapiens] | 6,04 | 3 |
| P35221-2 | Isoform 2 of Catenin alpha-1 [OS=Homo sapiens] | 5,52 | 4 |
| Q14974 | Importin subunit beta-1 [OS=Homo sapiens] | 5,46 | 7 |
| Q14517 | Protocadherin Fat 1 [OS=Homo sapiens] | 5,43 | 3 |
| Q9Y639-2 | Neuroplastin [OS=Homo sapiens] | 5,25 | 7 |
| P68371 | Tubulin beta-4B chain [OS=Homo sapiens] | 5,09 | 2 |

***Supplementary table 3: Mass spectrometry analysis of proteins in U87 derived EV.***

| **Accession** | **Description** | **Score Sequest HT** | **# PSMs** |
| --- | --- | --- | --- |
|  |  |  |  |
| P19823 | Inter-alpha-trypsin inhibitor heavy chain H2 [OS=Homo sapiens] | 2005,96 | 767 |
| P01023 | alpha-2-macroglobulin [OS=Homo sapiens] | 1157,1 | 723 |
| P07996 | thrombospondin-1 [OS=Homo sapiens] | 1102,42 | 451 |
| Q06033-1 | Inter-alpha-trypsin inhibitor heavy chain H3 [OS=Homo sapiens] | 841,63 | 352 |
| Q08380 | Galectin-3-binding protein [OS=Homo sapiens] | 688,68 | 261 |
| P02765 | Alpha-2-HS-glycoprotein [OS=Homo sapiens] | 652,94 | 262 |
| P26022 | Pentraxin-related protein PTX3 [OS=Homo sapiens] | 564,98 | 198 |
| P60709 | Actin, cytoplasmic 1 [OS=Homo sapiens] | 541,25 | 192 |
| P23142-4 | Isoform C of Fibulin-1 [OS=Homo sapiens] | 443,85 | 236 |
| P23142 | Fibulin-1 [OS=Homo sapiens] | 425,89 | 229 |
| P20742 | Pregnancy zone protein [OS=Homo sapiens] | 389,78 | 204 |
| P04264 | Keratin, type II cytoskeletal 1 [OS=Homo sapiens] | 370,02 | 156 |
| P04004 | Vitronectin [OS=Homo sapiens] | 327,77 | 101 |
| P62736 | Actin, aortic smooth muscle [OS=Homo sapiens] | 310,84 | 127 |
| P16112 | Aggrecan core protein [OS=Homo sapiens] | 309,78 | 120 |
| P04114 | apolipoprotein B-100 [OS=Homo sapiens] | 288,34 | 154 |
| P13611 | Versican core protein [OS=Homo sapiens] | 282,03 | 106 |
| Q2PPJ7-1 | Ral GTPase-activating protein subunit alpha-2 [OS=Homo sapiens] | 257,1 | 167 |
| P02771 | Alpha-fetoprotein [OS=Homo sapiens] | 232,17 | 78 |
| Q96Q89-2 | Isoform 2 of Kinesin-like protein KIF20B [OS=Homo sapiens] | 217,95 | 127 |
| O00468-7 | Isoform 7 of Agrin [OS=Homo sapiens] | 208,22 | 94 |
| P35527 | Keratin, type I cytoskeletal 9 [OS=Homo sapiens] | 192,33 | 96 |
| P16402 | Histone H1.3 [OS=Homo sapiens] | 189,57 | 85 |
| P01042 | kininogen-1 [OS=Homo sapiens] | 183,34 | 70 |
| P02452 | Collagen alpha-1(I) chain [OS=Homo sapiens] | 169,76 | 78 |
| P05556-1 | Integrin beta-1 [OS=Homo sapiens] | 162,43 | 83 |
| P19827-1 | Inter-alpha-trypsin inhibitor heavy chain H1 [OS=Homo sapiens] | 143,13 | 80 |
| P01024 | Complement C3 [OS=Homo sapiens] | 128,75 | 63 |
| P02751-15 | Isoform 15 of Fibronectin [OS=Homo sapiens] | 108,7 | 65 |
| P08697-1 | Alpha-2-antiplasmin [OS=Homo sapiens] | 98,21 | 38 |
| P26006-1 | Isoform 2 of Integrin alpha-3 [OS=Homo sapiens] | 85,13 | 45 |
| P0C0L4-1 | Complement C4-A [OS=Homo sapiens] | 73,36 | 34 |
| P00734 | Prothrombin [OS=Homo sapiens] | 61,2 | 21 |
| Q06828 | Fibromodulin [OS=Homo sapiens] | 61,03 | 34 |
| P12109 | Collagen alpha-1(VI) chain [OS=Homo sapiens] | 57,13 | 23 |
| O95497 | Pantetheinase [OS=Homo sapiens] | 50,73 | 24 |
| P68363 | Tubulin alpha-1B chain [OS=Homo sapiens] | 45,2 | 17 |
| P51884 | Lumican [OS=Homo sapiens] | 44,94 | 20 |
| P16070 | CD44 antigen [OS=Homo sapiens] | 43,7 | 21 |
| P36955 | Pigment epithelium-derived factor [OS=Homo sapiens] | 43,58 | 16 |
| O15230 | Laminin subunit alpha-5 [OS=Homo sapiens] | 42,24 | 31 |
| Q96CF2 | Charged multivesicular body protein 4c [OS=Homo sapiens] | 40,24 | 34 |
| P02649 | Apolipoprotein E [OS=Homo sapiens] | 36,93 | 15 |
| P21333 | Filamin-A [OS=Homo sapiens] | 36,29 | 21 |
| P35443 | Thrombospondin-4 [OS=Homo sapiens] | 34,95 | 30 |
| P07942 | Laminin subunit beta-1 [OS=Homo sapiens] | 34,05 | 26 |
| P08779 | Keratin, type I cytoskeletal 16 [OS=Homo sapiens] | 32,51 | 21 |
| Q5QNW6-2 | Isoform 2 of Histone H2B type 2-F [OS=Homo sapiens] | 31,43 | 24 |
| P02461 | Collagen alpha-1(III) chain [OS=Homo sapiens] | 30,83 | 27 |
| P68104 | Elongation factor 1-alpha 1 [OS=Homo sapiens] | 30,81 | 13 |
| P07195 | L-lactate dehydrogenase B chain [OS=Homo sapiens] | 30,12 | 16 |
| P23526-1 | Adenosylhomocysteinase [OS=Homo sapiens] | 26,44 | 15 |
| P49747 | Cartilage oligomeric matrix protein [OS=Homo sapiens] | 26,41 | 16 |
| P10124 | Serglycin [OS=Homo sapiens] | 26,35 | 19 |
| P05543 | thyroxine-binding globulin [OS=Homo sapiens] | 25,81 | 14 |
| Q9Y490 | Talin-1 [OS=Homo sapiens] | 23,43 | 10 |
| P00747 | Plasminogen [OS=Homo sapiens] | 23,3 | 12 |
| P98160 | Basement membrane-specific heparan sulfate proteoglycan core protein [OS=Homo sapiens] | 22,24 | 8 |
| P55290-4 | Isoform 4 of Cadherin-13 [OS=Homo sapiens] | 20,62 | 8 |
| Q16270-1 | Insulin-like growth factor-binding protein 7 [OS=Homo sapiens] | 18,49 | 9 |
| P11142-1 | Heat shock cognate 71 kDa protein [OS=Homo sapiens] | 16,56 | 9 |
| B9A064-1 | Immunoglobulin lambda-like polypeptide 5 [OS=Homo sapiens] | 16,47 | 8 |
| P10643 | Complement component C7 [OS=Homo sapiens] | 16,26 | 9 |
| P05156 | Complement factor I [OS=Homo sapiens] | 14,72 | 17 |
| P02753 | Retinol-binding protein 4 [OS=Homo sapiens] | 14,05 | 17 |
| P14618 | Pyruvate kinase PKM [OS=Homo sapiens] | 13,88 | 5 |
| Q15063-3 | Isoform 3 of Periostin [OS=Homo sapiens] | 13,65 | 11 |
| Q9NZR1 | Tropomodulin-2 [OS=Homo sapiens] | 13,35 | 7 |
| O75144-2 | Isoform 2 of ICOS ligand [OS=Homo sapiens] | 13,12 | 9 |
| P13640 | metallothionein-1G [OS=Homo sapiens] | 12,2 | 6 |
| P07900-2 | Isoform 2 of Heat shock protein HSP 90-alpha [OS=Homo sapiens] | 11,91 | 6 |
| P19320-1 | Vascular cell adhesion protein 1 [OS=Homo sapiens] | 11,48 | 4 |
| P00533-1 | epidermal growth factor receptor [OS=Homo sapiens] | 11,39 | 8 |
| Q15063-1 | Periostin [OS=Homo sapiens] | 11 | 10 |
| P13647 | keratin, type II cytoskeletal 5 [OS=Homo sapiens] | 10,05 | 9 |
| Q95604 | HLA class I histocompatibility antigen, Cw-17 alpha chain [OS=Homo sapiens] | 9,55 | 4 |
| Q15262-4 | Isoform 4 of Receptor-type tyrosine-protein phosphatase kappa [OS=Homo sapiens] | 9,51 | 5 |
| P05067-1 | Amyloid beta A4 protein [OS=Homo sapiens] | 8,39 | 4 |
| Q5JWF2-1 | Guanine nucleotide-binding protein G(S) subunit alpha isoforms XLas [OS=Homo sapiens] | 8,14 | 4 |
| P61224-1 | Ras-related protein Rap-1b [OS=Homo sapiens] | 7,88 | 7 |
| Q9BXJ4-3 | Isoform 3 of Complement C1q tumor necrosis factor-related protein 3 [OS=Homo sapiens] | 7,83 | 3 |
| P02786 | Transferrin receptor protein 1 [OS=Homo sapiens] | 7,61 | 3 |
| Q13885 | Tubulin beta-2A chain [OS=Homo sapiens] | 7,37 | 3 |
| Q12805 | EGF-containing fibulin-like extracellular matrix protein 1 [OS=Homo sapiens] | 7,36 | 4 |
| P08123 | Collagen alpha-2(I) chain [OS=Homo sapiens] | 7,31 | 7 |
| P26038 | Moesin [OS=Homo sapiens] | 6,58 | 3 |
| P02774-3 | Isoform 3 of Vitamin D-binding protein [OS=Homo sapiens] | 6,56 | 3 |
| P0DMV8 | heat shock 70 kDa protein 1A [OS=Homo sapiens] | 6,2 | 3 |
| P01009-1 | alpha-1-antitrypsin [OS=Homo sapiens] | 5,99 | 3 |
| P13726-1 | tissue factor [OS=Homo sapiens] | 5,09 | 2 |

***Supplementary table 4: Mass spectrometry analysis of proteins in MDA-MB-231 derived EV.***
